# New marine gastropod records for the Hellenic waters

**DOI:** 10.1186/s40709-018-0077-3

**Published:** 2018-03-20

**Authors:** Thanasis Manousis, Constantinos Kontadakis, Georgios Polyzoulis, George Mbazios, Sofia Galinou-Mitsoudi

**Affiliations:** 1 PO Box 48 K, 57500 Epanomi, Greece; 2 Aristotelous 24, Hellinicon, 16777 Athens, Greece; 3 PO Box 891, 57001 N. Redestos, Greece; 4 Orfanidou 27, 11142 Athens, Greece; 5 Department of Civil Engineering, School of Technological Applications, Alexander Technological Educational Institute of Thessaloniki (A.T.E.I.Th.), PO Box 141, 574 00 Thessaloniki, Greece

**Keywords:** Biodiversity, Gastropods, Mediterranean Sea, Greece

## Abstract

**Background:**

The Hellenic Seas are influenced by on-going environmental changes and the introduction of alien species, which are expected to have an impact on their biodiversity. This study contributes to the knowledge of the Hellenic marine gastropod biodiversity, expanding data over the entire Greek territory, during the period from October 2008 to March 2017.

**Results:**

This work presents 45 species of gastropods not previously reported from Greece or reported only once, belonging to 19 families. From those species, one (*Horologica* sp.) is, most probably, an undescribed species, 17 are new for the Eastern Mediterranean Sea and 40 are new for the Hellenic fauna. Main taxonomic characteristics and ecological information such as habitat, distribution and origin, are given and discussed.

**Conclusions:**

By this report, the Hellenic gastropod biodiversity is enriched by 40 new records, out of which, 17 are new for the Eastern Mediterranean Sea, 4 are Lessepsian migrants previously reported for the Mediterranean Sea and 1 is probably a new species.

## Background

The Mediterranean Sea is rich in biodiversity. Almost two decades ago, about 8500 species have been estimated to occur [1]; this number doubled recently to more than 17,000 species [2]. This impressive increase is attributed to (a) the rising number of relevant studies in new areas and at different depths and biotopes, (b) the introduction of alien species, which reached almost 1000 species by 2012 [2], and (c) the increasing rate of modification of the Mediterranean Sea ecosystems [3]. The environmental changes that have influenced the biodiversity during the last three decades, the reasons for the occurrence of aliens, the frequency of the records, the vectors and the distribution pathways have been extensively discussed [2, 4–12].

The Hellenic Seas, as a part of the Eastern Mediterranean Sea, have been influenced by the on-going environmental changes, and Aegean Sea, in particular, after a long and slow cooling period from the late sixties to the early nineties, started to warm rapidly [13]. As a result, dense waters from the Adriatic Sea shifted to the Aegean Sea, a phenomenon known as the “Eastern Mediterranean Transient” [14, 15]. These environmental changes combined with the effects of navigation and sea currents, were expected to have an impact on the biodiversity. Indeed, recent publications revealed new species for the Hellenic Seas [16–22]. The aim of this study was to further contribute to the knowledge of the Gastropod fauna of the Hellenic Seas.

## Results

### The records

Among the 226 specimens collected, 45 species were identified belonging to 19 families and are listed with data on their habitat, mode of life and origin in Table 1. Among them, 4 are recognized as already known for the Mediterranean Sea Lessepsian migrants, 17 as new for the Eastern Mediterranean Sea fauna, and 40 as new for the Hellenic fauna (collection stations are indicated in the map of Fig. 1 while the species are presented in Figs. 2, 3, 4, 5, 6, 7, 8, 9, 10, 11, 12, 13, 14, 15, 16, 17, 18, 19, 20, 21).

**Table 1:** Gastropod records, stations, habitat and distribution details (in the study area)

Family	Species	New records	The collection stations			Zone/depth (m)	Habitat	Mode of life [107]	Found	Origin
			N Aegean Sea	S Aegean Sea	Ionian Sea					
Fissurellidae	*Diodora funiculata* (Reeve, 1850)	R1		14		1	Rocky bottom with *Mytilus galloprovincialis*	Herbivorous	Alive	E Mediterranean Sea Lessepsian
	*Fissurisepta granulosa* Jeffreys, 1883	R1, EM	1			400	*Coralliferous* bottom	Herbivorous	Shell	W & C Mediterranean Sea
Lepetidae	*Iothia fulva* (O. F. Müller, 1776)	R1	1			400	*Coralliferous* bottom	Detritivorous	Shells	Mediterranean Sea
	*Propilidium exiguum* (W. Thompson, 1844)	R1	1			400	*Coralliferous* bottom	Probably detritivorous	Shells	Mediterranean Sea
Anatomidae	*Anatoma tenuisculpta* (Seguenza, 1880)	R1, EM	1			400	*Coralliferous* bottom	Herbivorous	Shells	W Mediterranean Sea
Addisoniidae	*Addisonia excentrica* (Tiberi, 1855)	R1	1			50	Mixed bottom	Herbivorous	Shell	Mediterranean Sea
Skeneidae	*Akritogyra conspicua* (Monterosato,1880)	R1	1			400	*Coralliferous* bottom	No documentation on diet	Shells	W Mediterranean Sea
	*Cirsonella romettensis* (Granata-Grillo, 1877)	R1, EM	1			400	*Coralliferous* bottom	No documentation on diet	Shells	W Mediterranean Sea
	*Lissomphalia bithynoides* (Monterosato, 1880)	R1			27	80	Mixed bottom	No documentation on diet	Shell	Mediterranean Sea
Chilodontidae	*Danilia costellata* (O.G. Costa, 1861)	R1		16, 22, 23, 24		80, 120	Coralliferous bottom and maerl	Herbivorous	Alive & shell	Mediterranean Sea
Trochidae	*Gibbula vimontiae* Monterosato, 1884	R1, EM		18		40	Mixed bottom	Herbivorous	Alive	W Mediterranean Sea
Neritidae	*Smaragdia souverbiana* (Montrouzier in Souverbie & Montrousier, 1863)	R2		14, 17, 23		0, 30, 40	Mixed bottom	Herbivorous	Alive & shells	E Mediterranean Sea Lessepsian
Cerithiidae	*Rhinoclavis kochi* (Philippi, 1848)	R1		19, 20		1–3	*Sandy bottom*	Herbivorous	Shells	E Mediterranean Sea Lessepsian
Newtoniellidae	*Cerithiella metula* (Lovén, 1846)	R1	1			400	*Coralliferous* bottom	Feeds on sponges	Shells	Mediterranean Sea
Cerithiopsidae	*Cerithiopsis annae* Cecalupo & Buzzurro, 2005	R1, EM	5			8	*Aplysina aerophoba*	Feeds on sponges	Alive	C Mediterranean Sea
	*Cerithiopsis buzzurroi* (Cecalupo & Robba, 2010)	R1	5	20, 23		8–90	Mixed bottom	Feeds on sponges	Alive & shell	Mediterranean Sea
	*Cerithiopsis denticulata* (Cecalupo & Robba, 2010)	R1	5, 6, 9	23		7, 15, 50	*Aplysina aerophoba* & *Euspongia officinalis*	Feeds on sponges	Shell	Mediterranean Sea
	*Cerithiopsis jeffreysi* Watson, 1885	R1	6	25		3	*Aplysina aerophoba*	Feeds on sponges	Alive & shell	Mediterranean Sea
	*Cerithiopsis ladae* Prkic & Buzzurro, 2007	R1, EM	7, 9			4, 40	*Aplysina aerophoba*, *Sarcotragus fasciculatus*	Feeds on sponges	Alive	W & C Mediterranean Sea
	*Cerithiopsis oculisfictis* Prkic & Mariottini, 2010	R1, EM	9			10	*Cladocora caespitosa*	Feeds on sponges	Alive	C Mediterranean Sea
	*Cerithiopsis petanii* Prkic & Mariottini, 2010	R1, EM	9			10	Mixed bottom	Feeds on sponges	Alive	C Mediterranean Sea
	*Cerithiopsis pulvis* (Issel, 1869)	R1		20, 21		10	Mixed bottom	Feeds on sponges	Shells	E Mediterranean Sea Lessepsian
	*Cerithiopsis scalaris* Locard, 1891	R1	9, 10	23		10, 60	*Muddy* bottom Mixed bottom	Feeds on sponges	Alive & shells	Mediterranean Sea
	*Horologica* sp.	R1, EM, M	13			6	*Aplysina aerophoba*	Feeds on sponges	Alive	Unknown
	*Krachia tiara* (Monterosato 1874)	R1		21		80	Mixed bottom	Feeds on sponges	Shell	Mediterranean Sea
Newtoniellidae	*Cerithiella metula* (Lovén, 1846)	R1	1			400	*Coralliferous* bottom	Feeds on sponges	Shells	Mediterranean Sea
Triphoridae	*Monophorus amicitiae* Romani, 2015	R1, EM		23		70, 90	Maerl	Feeds on sponges	Shells	W Mediterranean Sea
	*Obesula marisnostri* *Bouchet, 1985*	R2 (data for the animal)		23		100	Maerl	Feeds on sponges	Alive	Mediterranean Sea
	*Strobiligera brychia* (Bouchet & Guillemot, 1978)	R1, EM	1			400	Rocky bottom	Feeds on sponges	Shells	W Mediterranean Sea
Epitoniidae	*Epitonium tryoni* (de Boury, 1913)	R1, EM			26	200	Rocky bottom	Feeds on anthozoa	Shell	W Mediterranean Sea
	*Janthina pallida* W. Thompson, 1840	R1			28	Beached	Sea surface	Feeds on cnidaria	Shells	Cosmopolitan
	*Opalia crenata* (Linnaeus, 1758)	R2			29	Beached 5–10	Rocky bottom	Feeds on anthozoa	Shells	Mediterranean Sea
Nystiellidae	*Narrimania concinna* (Sykes, 1925)	R1, EM	11	23		90, 135	Hard substrate	Feeds on sponges	Alive & shell	S Mediterranean Sea
	*Opaliopsis atlantis* (Clench & Turner, 1952)	R1, EM	1, 2			200, 400	Rocky and *coralliferous*	Feeds on anthozoa	Alive & shells	Mediterranean Sea
Eulimidae	*Acrochalix* cf. *callosa* Bouchet & Warén, 1986	R1, EM, M	1, 10			70, 450	Coralliferous bottom Muddy bottom	Feeds on echinodermata	Shells	NE Atlantic
	*Campylorhaphion* cf. *famelicum* (Watson, 1883)	R1	4			35	Mixed bottom	Feeds on echinodermata	Alive	Mediterranean Sea
	*Curveulima dautzenbergi* (Pallary, 1900)	R1, EM	7, 9			40	Mixed bottom	Feeds on echinodermata	Alive	W Mediterranean Sea
	*Haliella stenostoma* (Jeffreys, 1858)	R1	1			400	*Coralliferous* bottom	Feeds on echinodermata	Shell	Mediterranean Sea
	*Nanobalcis nana* (Monterosato, 1878)	R1	3	23		40–120	Mixed bottom	Feeds on echinodermata	Alive & shells	Mediterranean Sea
	*Sticteulima jeffreysiana* (Brusina, 1869)	R2	3, 4, 5, 12, 13	23		30–70	Mixed bottom	Feeds on echinodermata	Alive & shells	Mediterranean Sea
Muricidae	*Aspella anseps* (Lamarck, 1822)	R1		21		6–10	Mixed bottom	Benthic predator	Shells	Mediterranean Sea
	*Nucella lapillus* (Linnaeus, 1758)	R1, EM	8			10	Mixed bottom	Carnivorous	Alive	W & C Mediterranean
Columbellidae	*Mitrella pallaryi* (Dautzenberg, 1927)	R1		23		30-70	Biogenic substrate	Carnivorous	Alive	Mediterranean Sea
Architectonicidae	*Heliacus jeffreysianus* (Tiberi, 1867)	R2		16		100	Maerl	Cnidarian ectoparacite	Alive	Mediterranean Sea
	*Spirolaxis clenchi* Jaume & Borro, 1946	R1	1			400	*Coralliferous bottom*	Cnidarian ectoparacite	Shells	Mediterranean Sea
Mathildidae	*Mathilda coronata* Monterosato, 1875	R1		17		80–120	Biogenic substrate Maerl	Feeds on cnidaria	Shells	Mediterranean Sea

R1 first record for Greece; R2 second record for Greece; EM first record for the Eastern Mediterranean Sea; M first record for the Mediterranean Sea

**Fig. 1:**
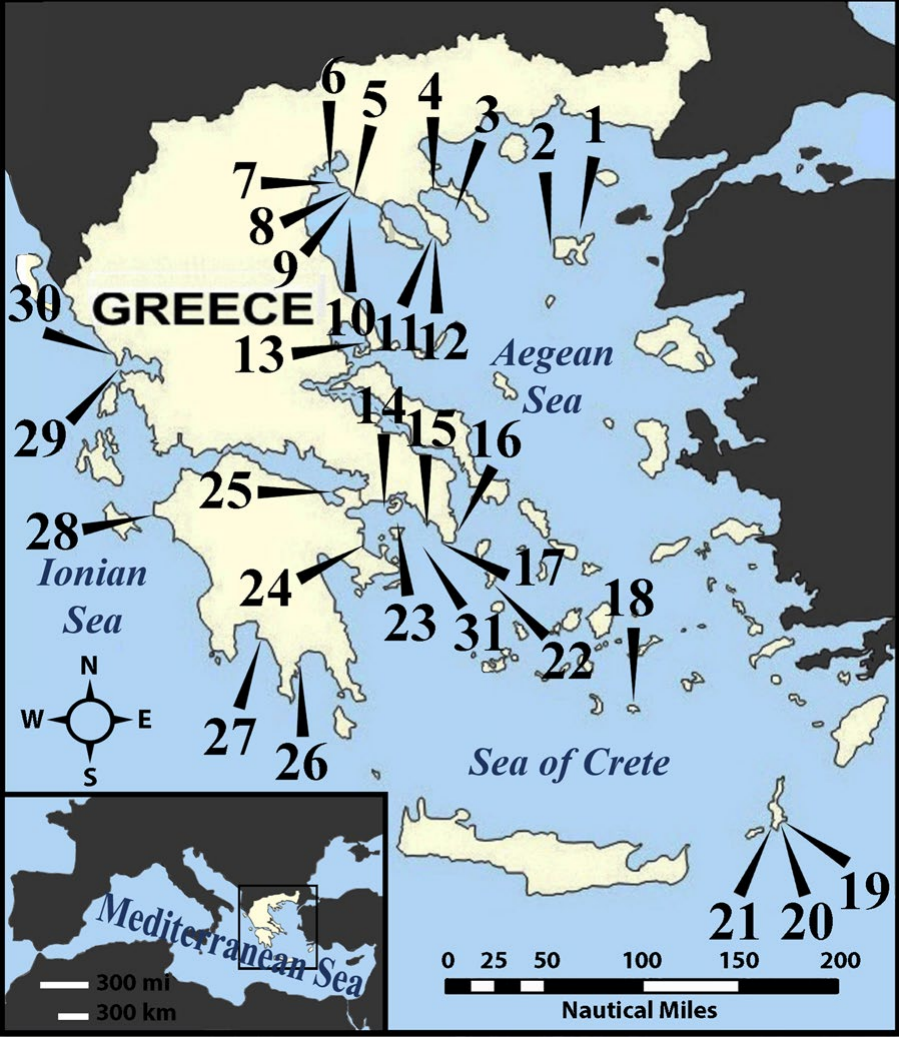
Map of collection stations for the species reported in this study. Stations in North Aegean Sea: 1. Limnos Island; 2. Kaspakas, Limnos Island; 3. Central Siggitikos Gulf; 4. Pyrgadikia, Chalkidiki; 5. Cape, Epanomi; 6. Aggelohori; 7. Nea Michaniona; 8. Palioura, Epanomi; 9. Paralia, Epanomi; 10. Central Thermaikos Gulf; 11. Toroni, Chalkidiki; 12. Tristinika, Chalkidiki; 13. Chorto, Pagasitikos Gulf. Stations in South Aegean Sea: 14. Eandio, Salamina Island, 15. Anavissos, Attiki; 16. Lavrio, Attiki; 17. Legrena, Attiki; 18. Anafi, Island; 19. Damatria, Karpathos Island; 20. Diakoftis, Karpathos Island; 21. Amoopi, Karpathos Island; 22. Kythnos, Island; 23. Central Saronikos Gulf; 24. Epidaurus Gulf; 25. Ireo, Korynthiakos Gulf. Stations in the Ionian Sea: 26. Gythio, Lakonikos Gulf; 27. Kardamili, Messenian Gulf; 28. Castro, Kyllini; 29. Kanali, Preveza; 30. Pantokratoras, Preveza. 31. South Saronikos Gulf

**Fig. 2:**
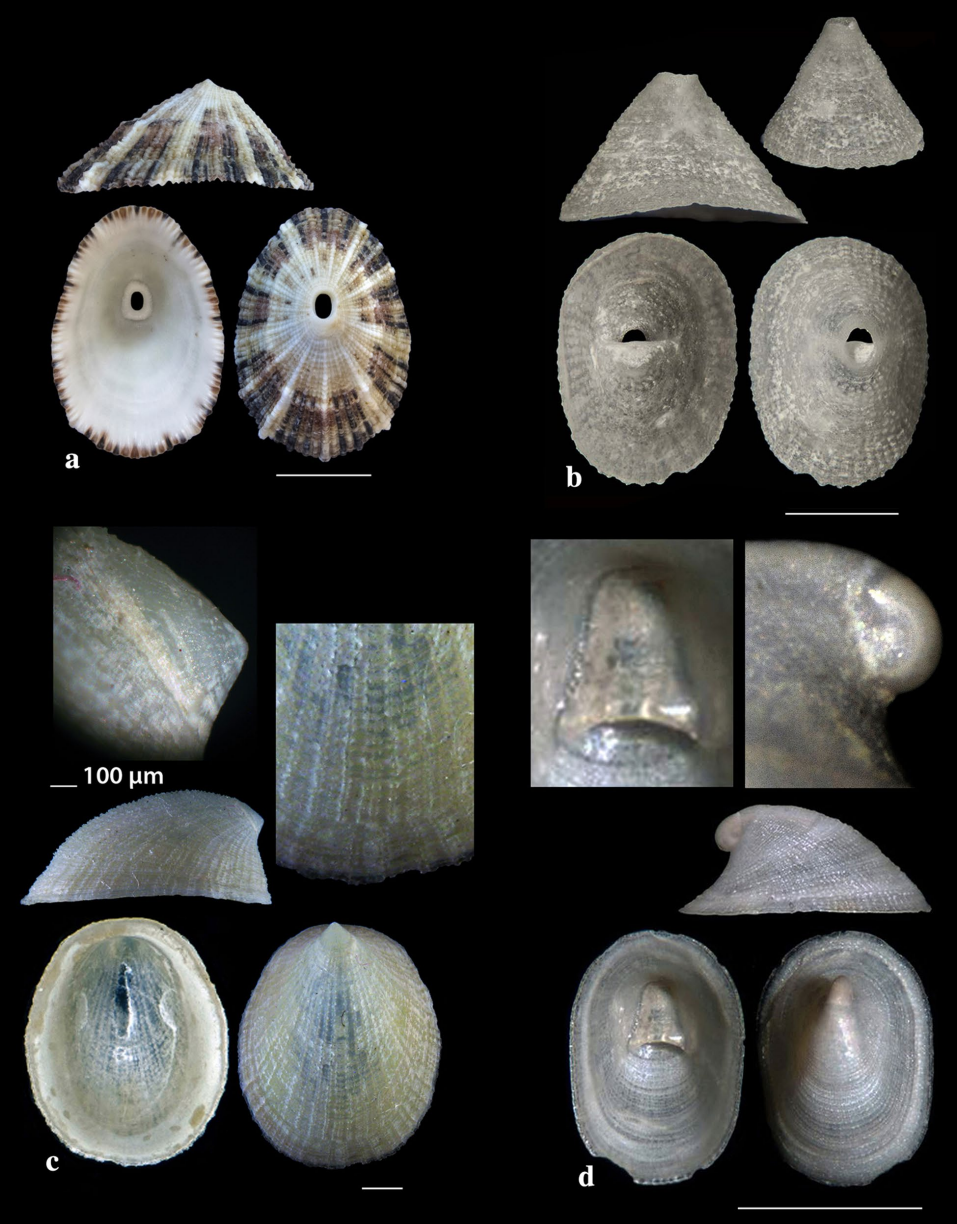
**a** *Diodora funiculata*, **b** *Fissurisepta granulosa*, **c** *Iothia fulva*, **d** *Propilidium exiguum*. Bar = 1 mm, unless otherwise indicated

**Fig. 3:**
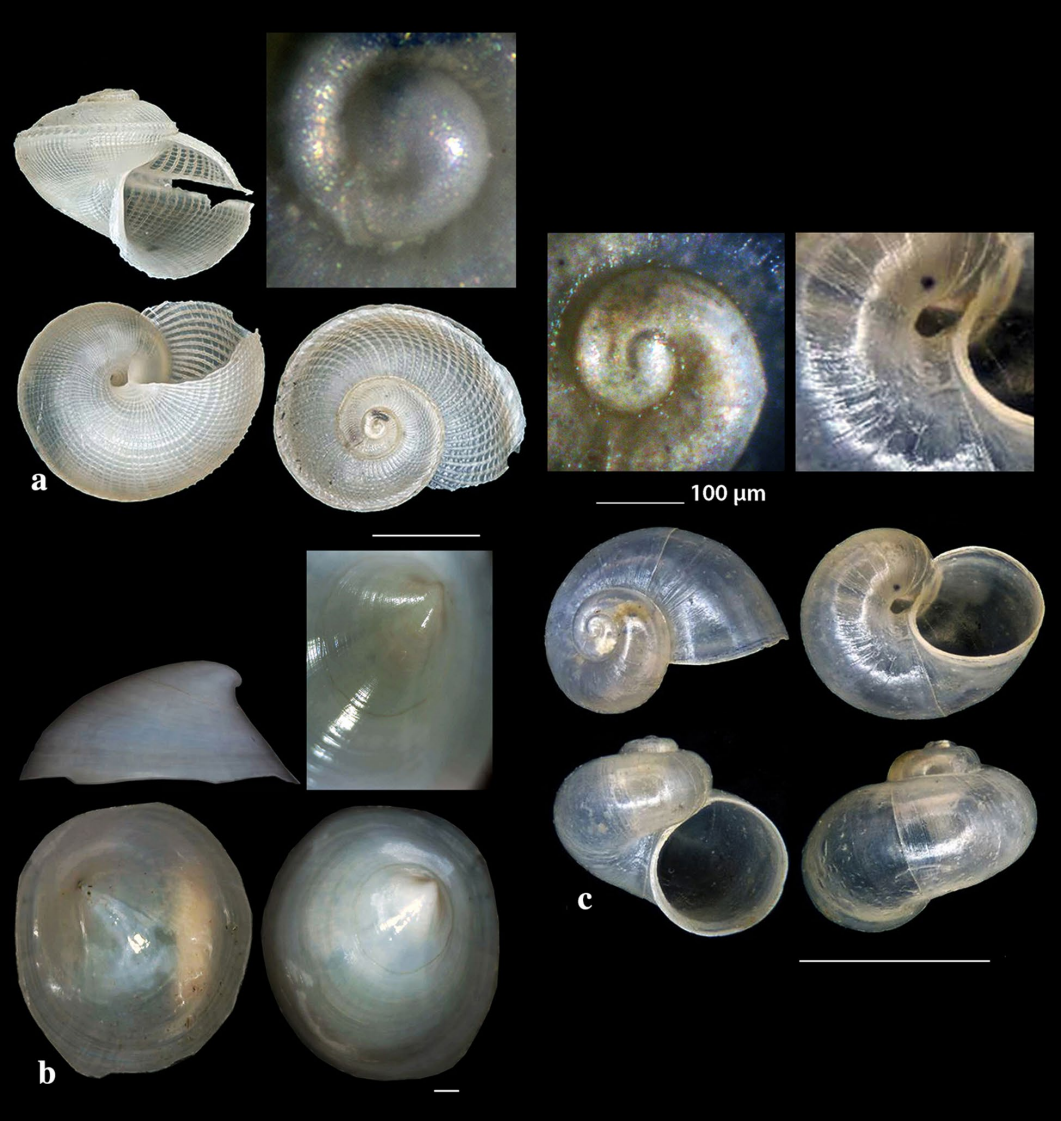
**a** *Anatoma tenuisculpta*, **b** *Addisonia excentrica*, **c** *Akritogyra conspicua*. Bar = 1 mm

**Fig. 4:**
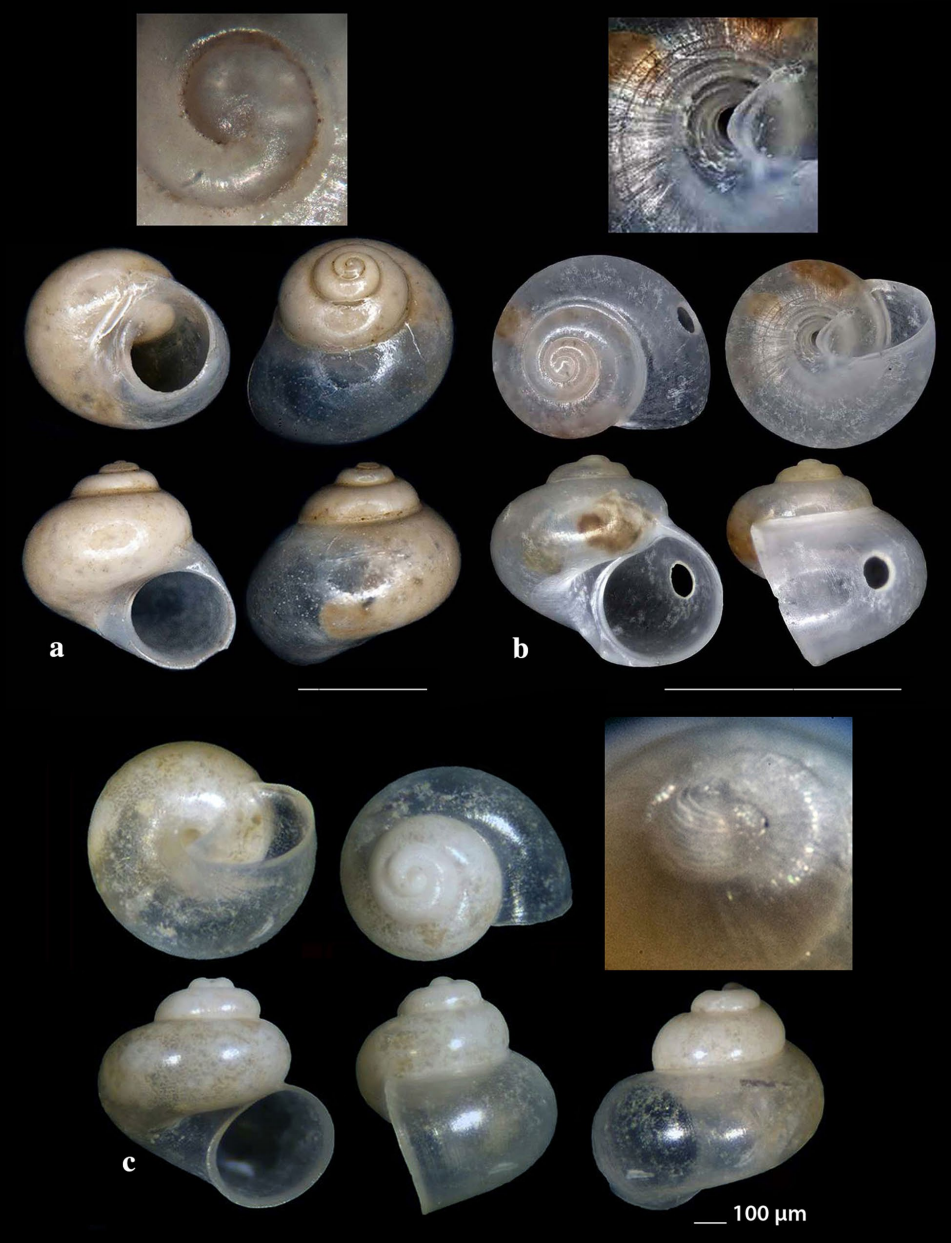
**a**, **b** *Cirsonella romettensis*, **c** *Lissomphalia bithynoides.* Bar = 1 mm, unless otherwise indicated

**Fig. 5:**
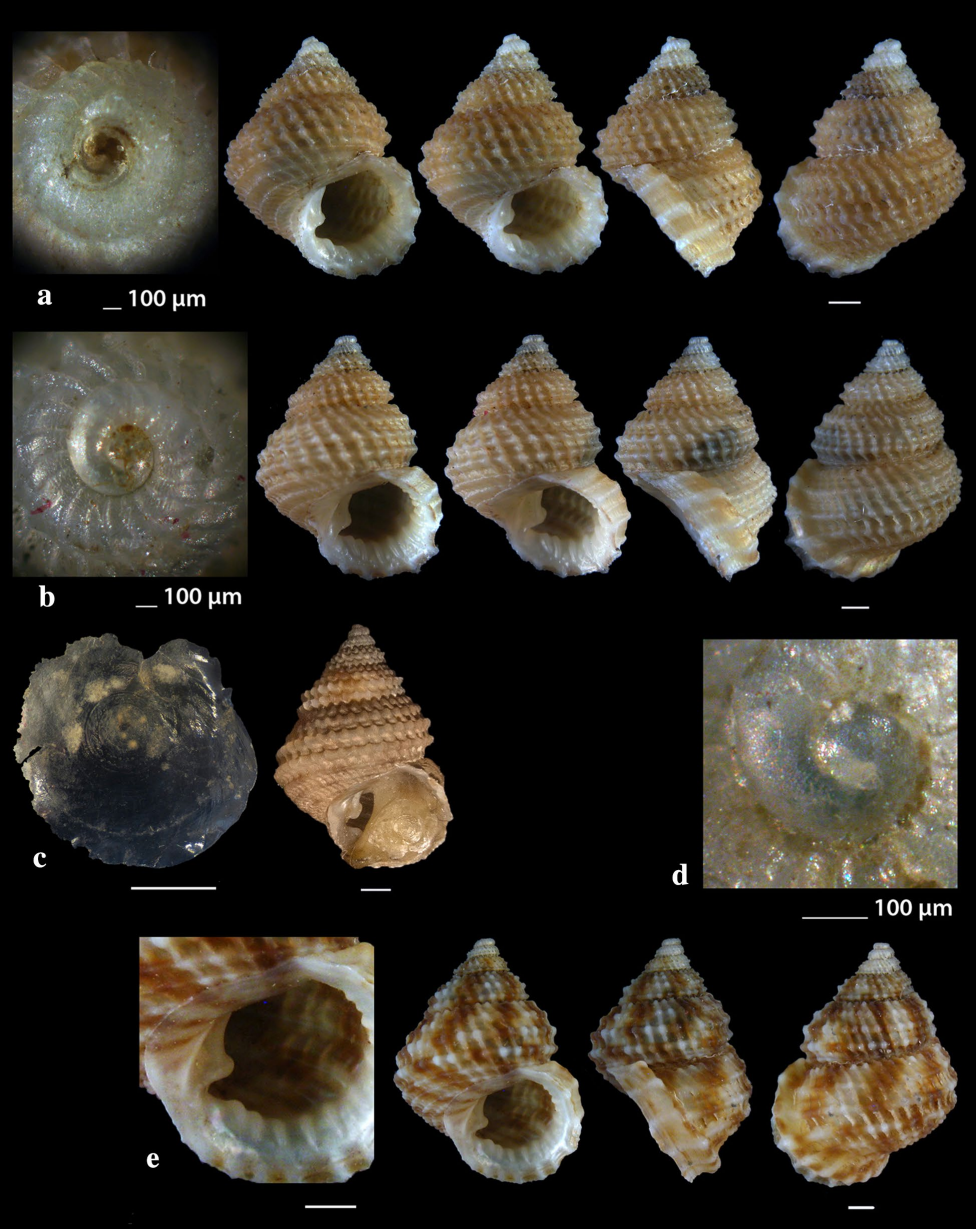
**a**–**c** *Danilia costellata*, **d**–**e** *Danilia tinei.* Bar = 1 mm, unless otherwise indicated

**Fig. 6:**
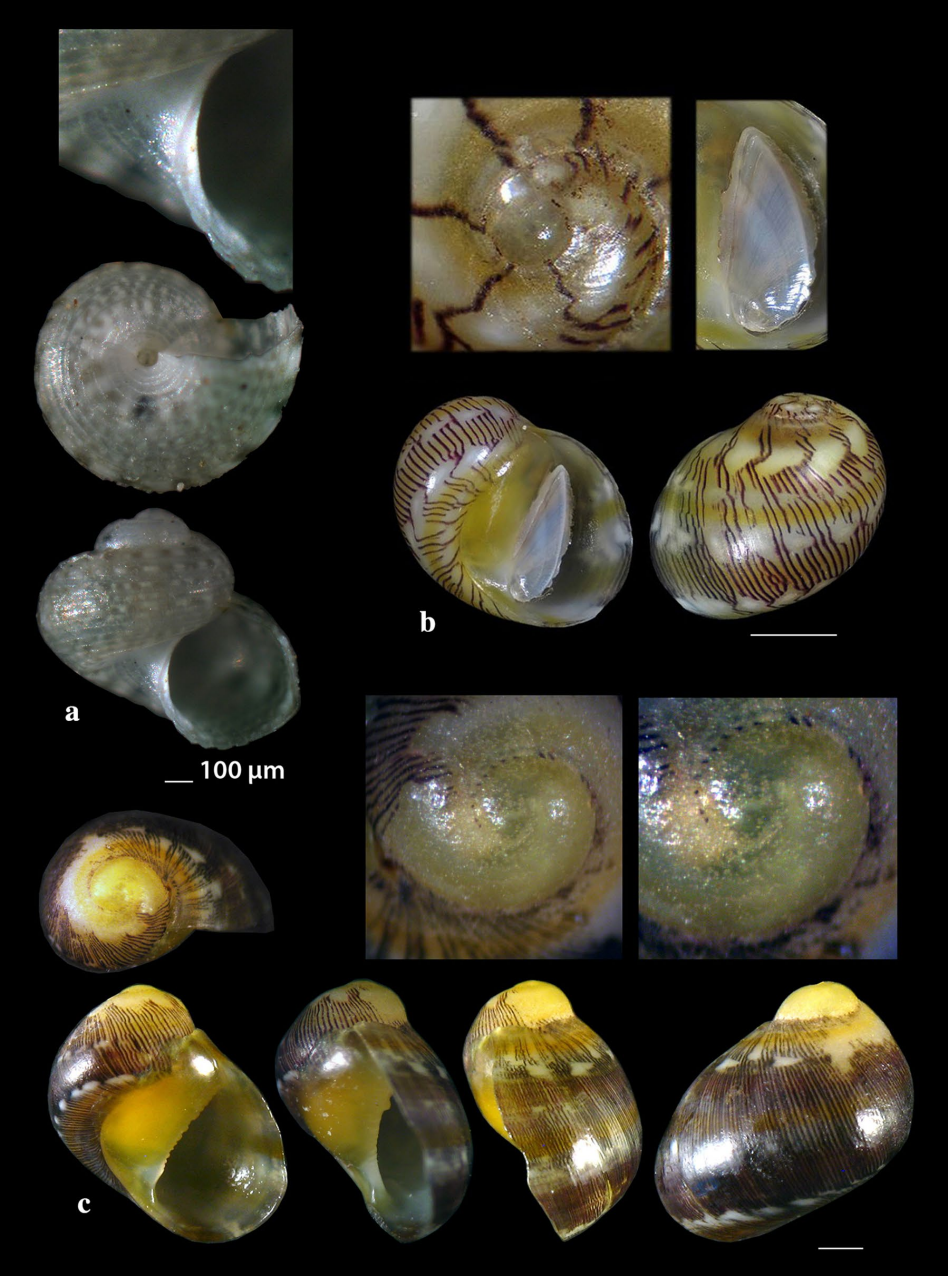
**a** *Gibbula* cf. *vimontiae*, **b**, **c** *Smaragdia souverbiana.* Bar = 1 mm, unless otherwise indicated

**Fig. 7:**
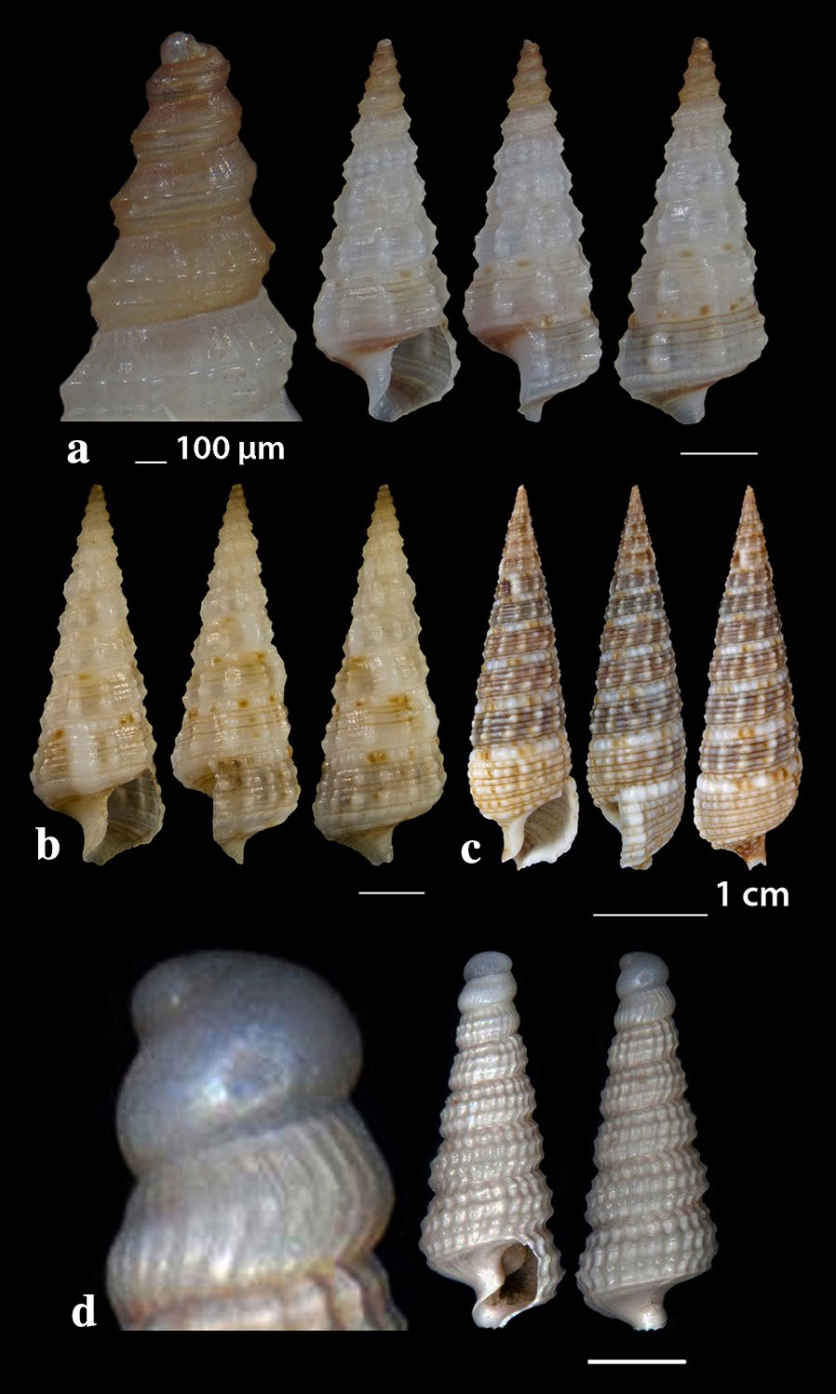
**a**–**c** *Rhinoclavis kochi*, **d** *Cerithiella metula.* Bar = 1 mm, unless otherwise indicated

**Fig. 8:**
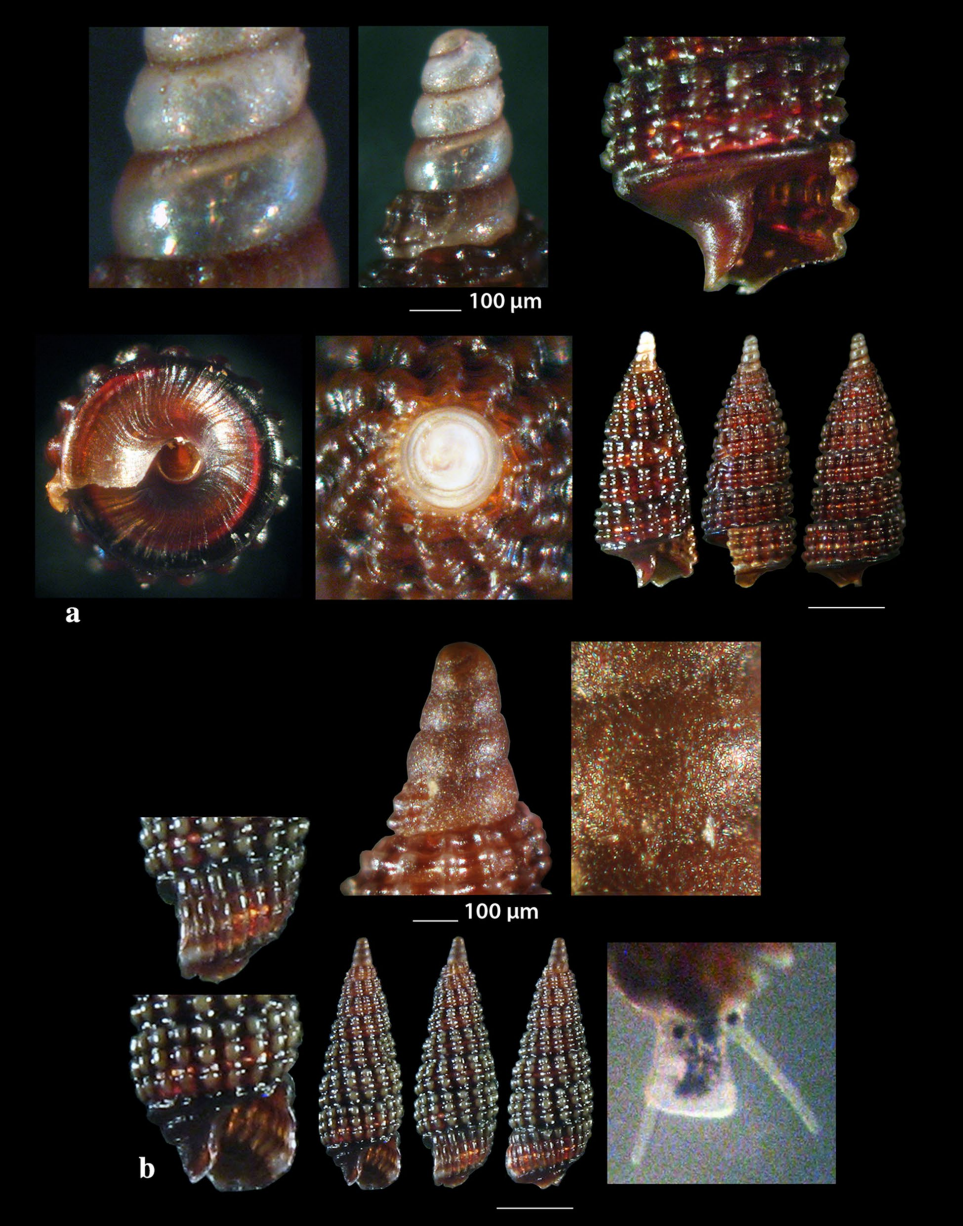
**a** *Cerithiopsis annae*, **b** *Cerithiopsis tubercularis.* Bar = 1 mm, unless otherwise indicated

**Fig. 9:**
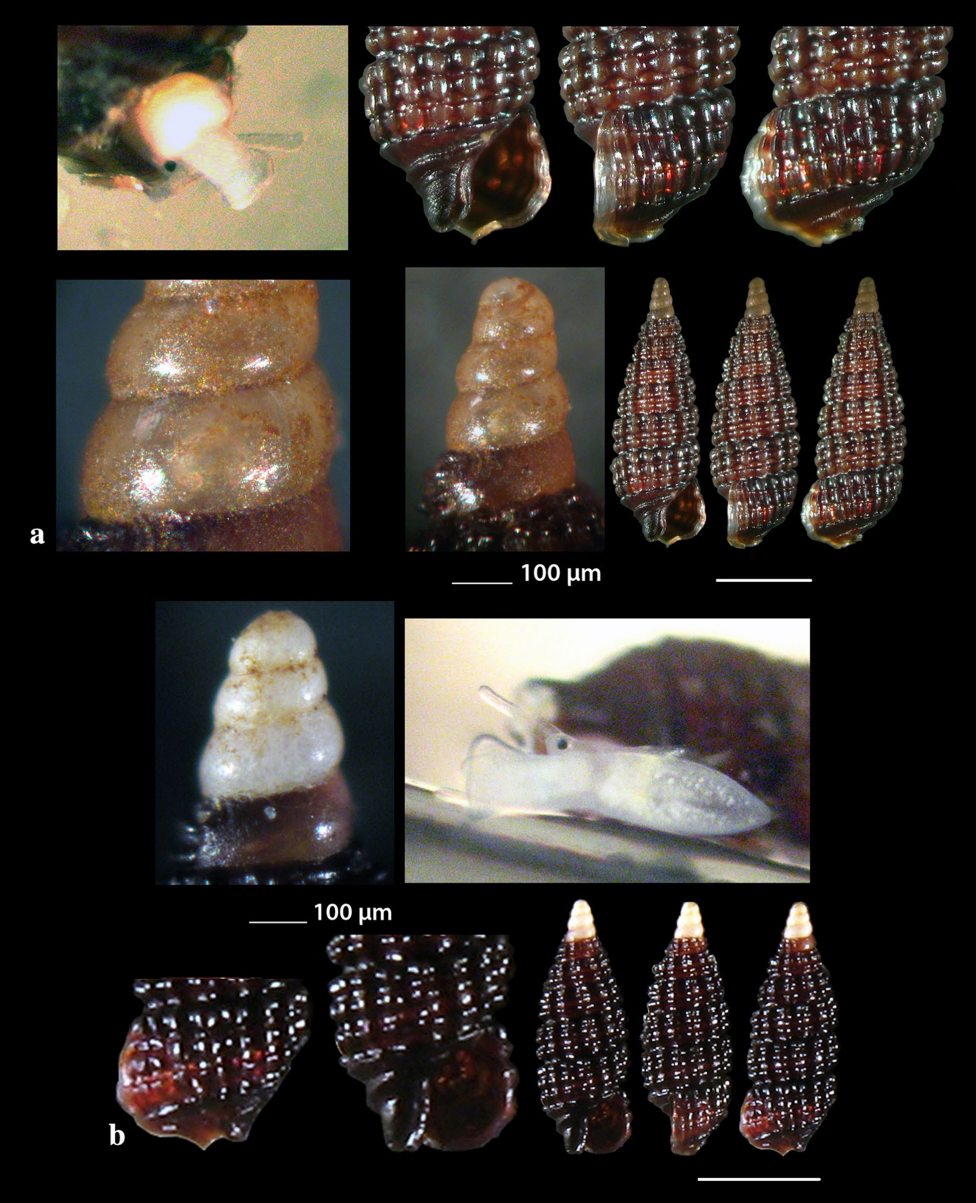
**a** *Cerithiopsis nana*, **b** *Cerithiopsis minima*. Bar = 1 mm, unless otherwise indicated

**Fig. 10:**
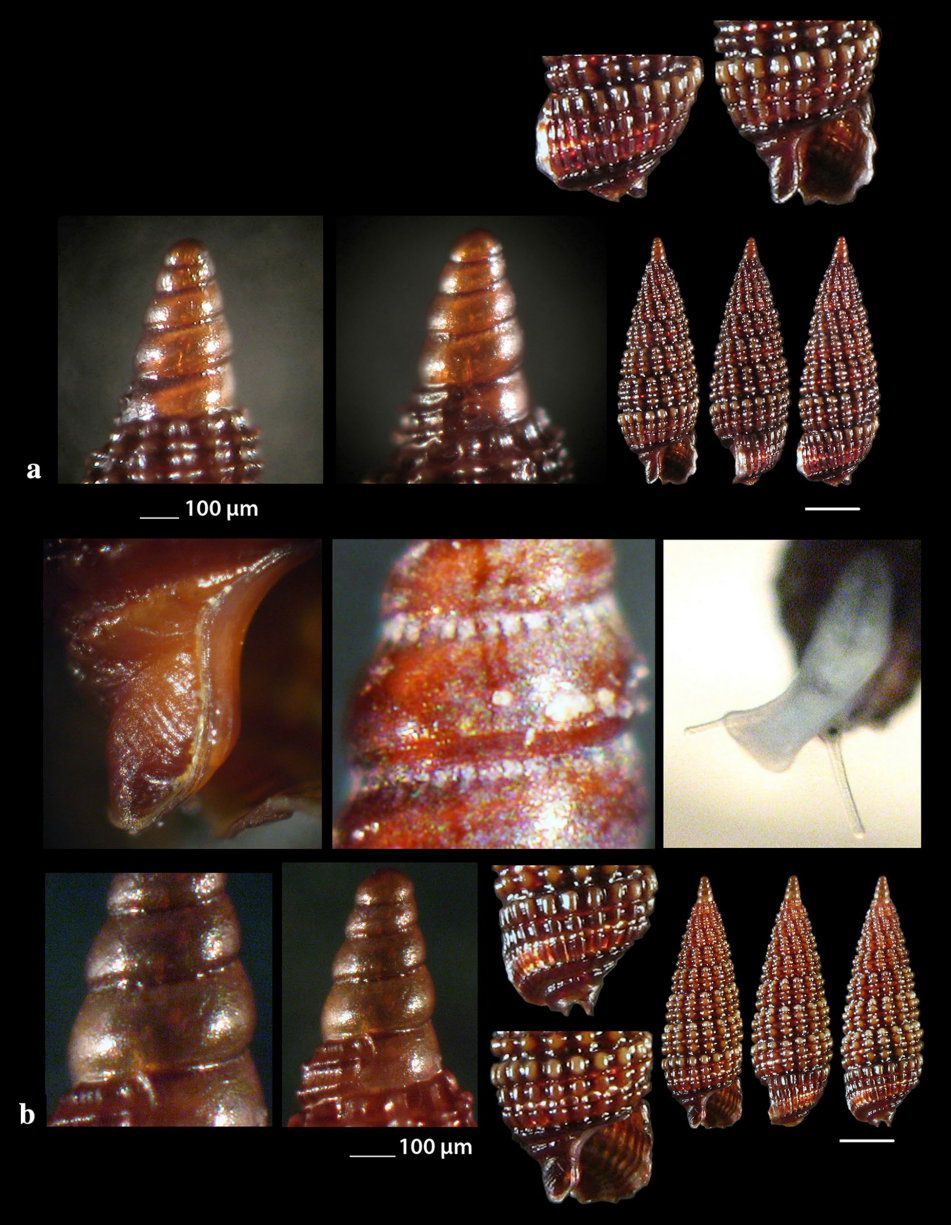
**a**, **b** *Cerithiopsis buzzurroi*. Bar = 1 mm, unless otherwise indicated

**Fig. 11:**
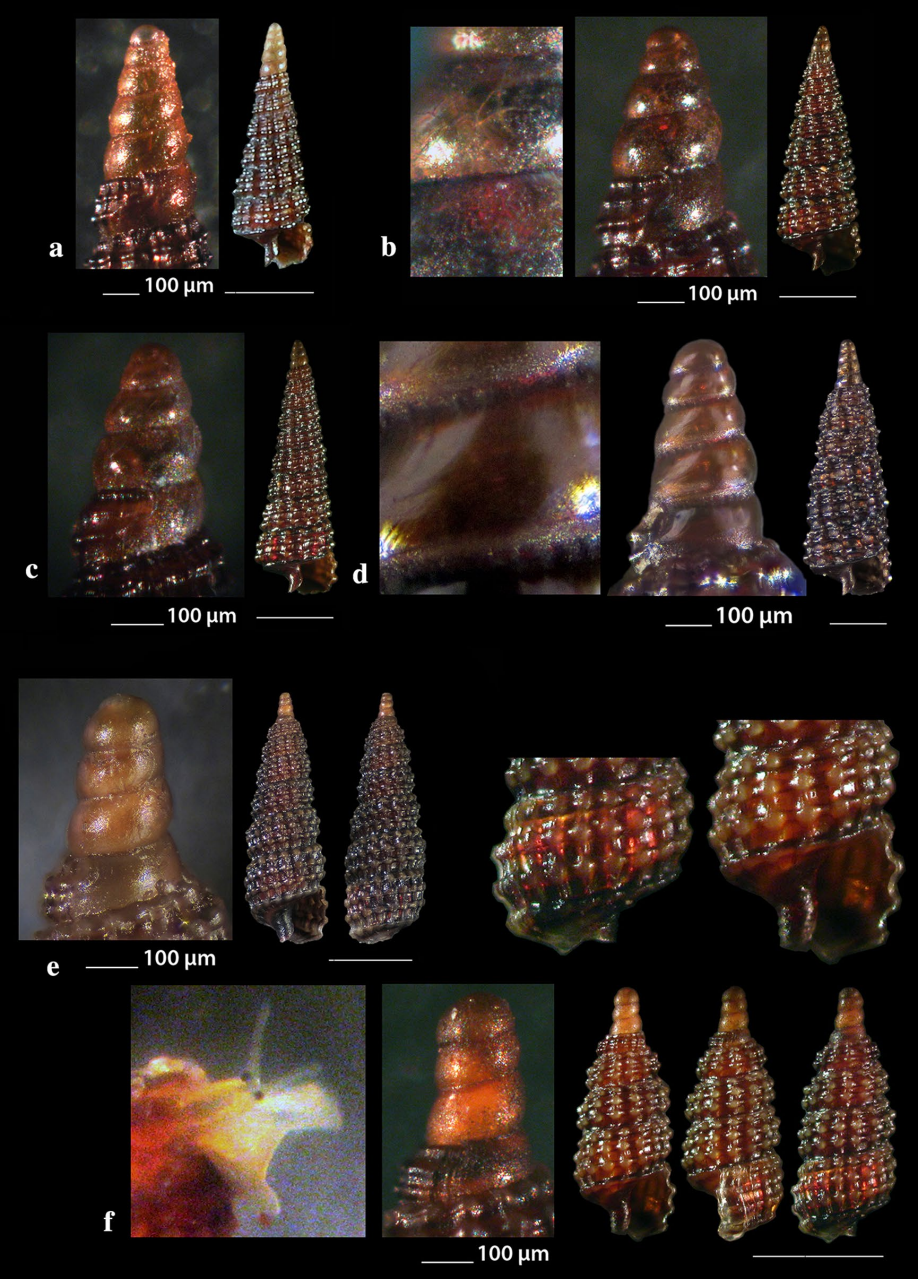
**a**–**d** *Cerithiopsis denticulata*, **e**, **f** *Cerithiopsis jeffreysi*. Bar = 1 mm, unless otherwise indicated

**Fig. 12:**
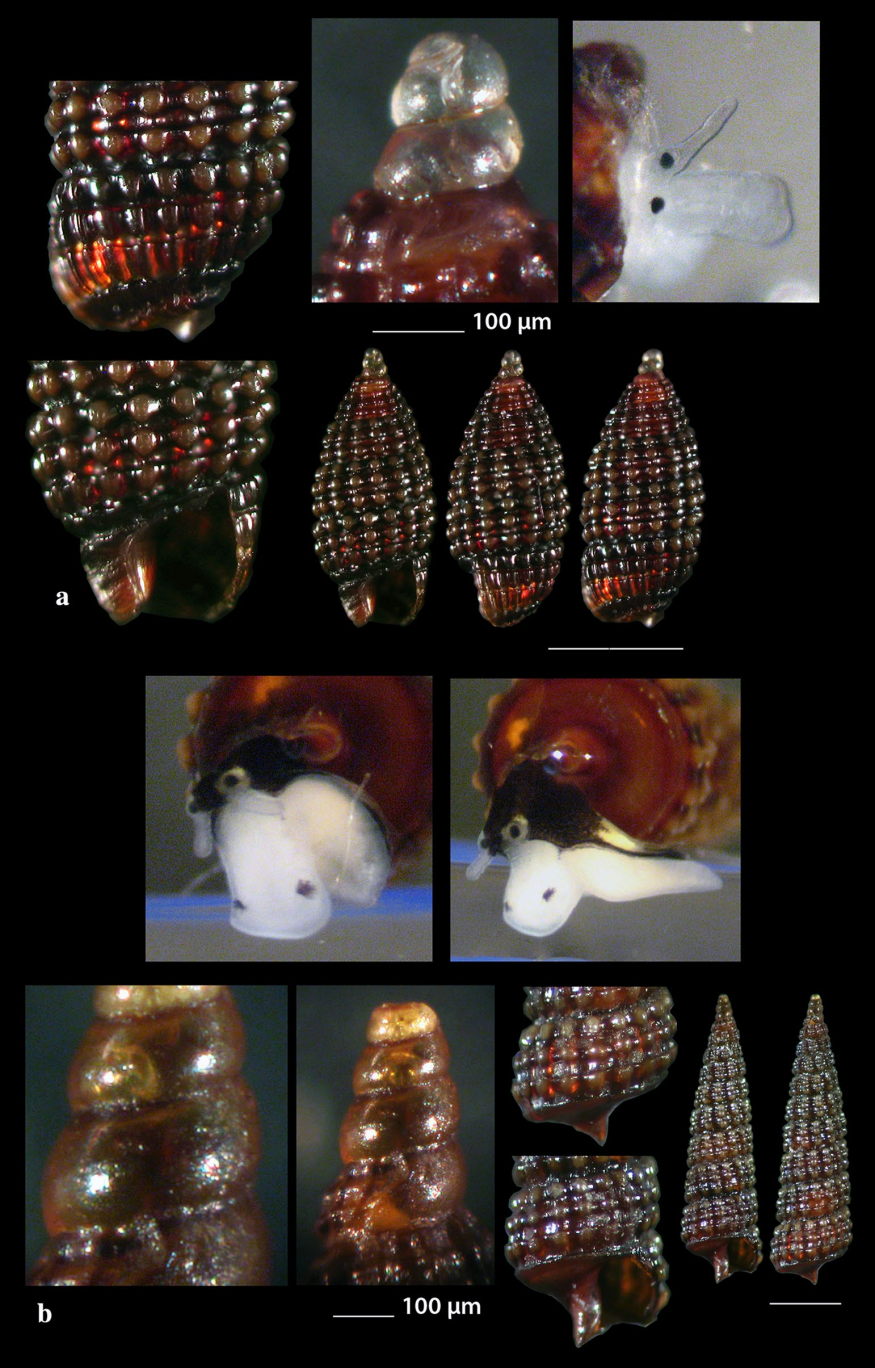
**a** *Cerithiopsis ladae*, **b** *Cerithiopsis oculisfictis*. Bar = 1 mm, unless otherwise indicated

**Fig. 13:**
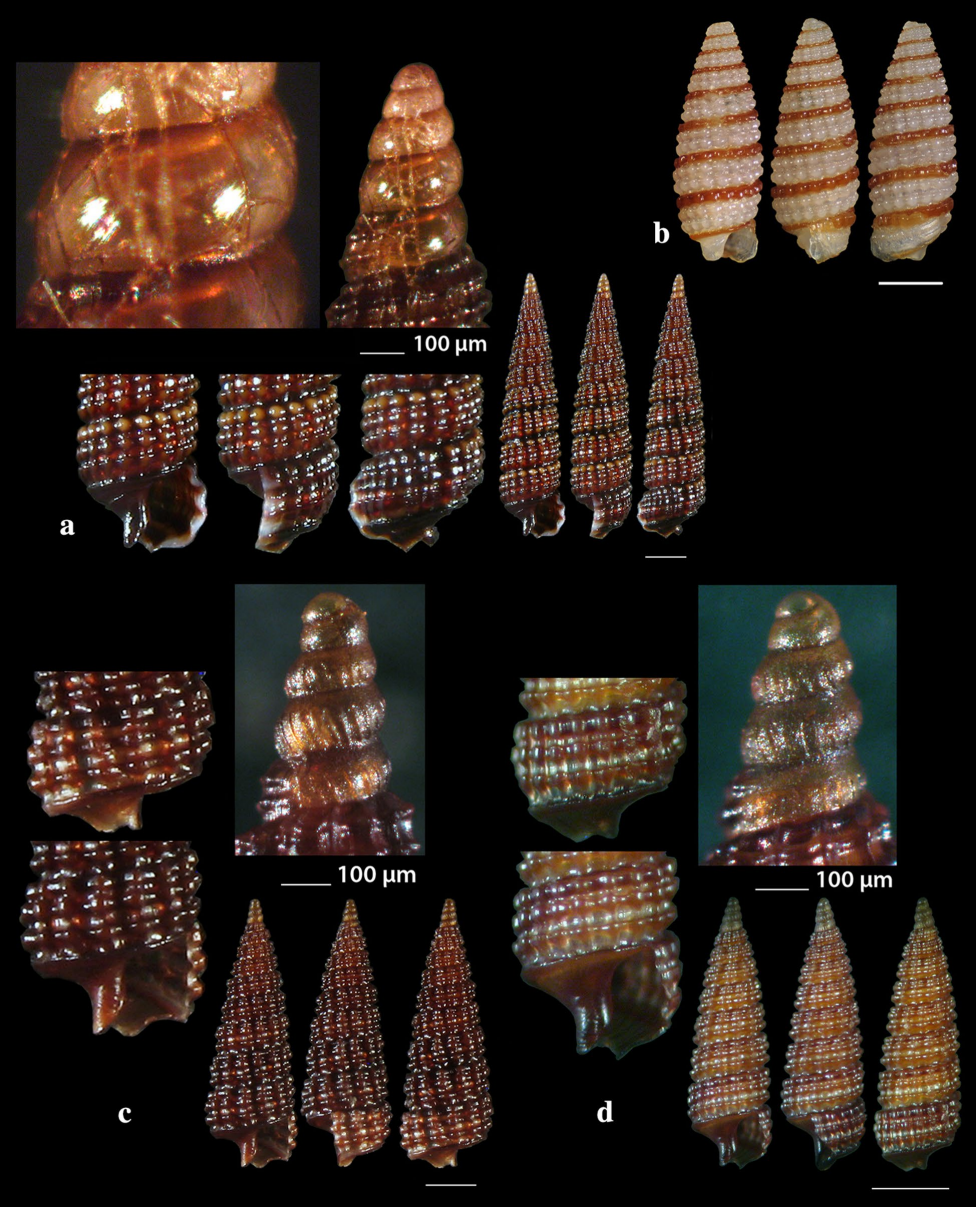
**a** *Cerithiopsis petanii*, **b** *Cerithiopsis pulvis*, **c** *Cerithiopsis scalaris*, **d** *Cerithiopsis* cf. *scalaris*. Bar = 1 mm, unless otherwise indicated

**Fig. 14:**
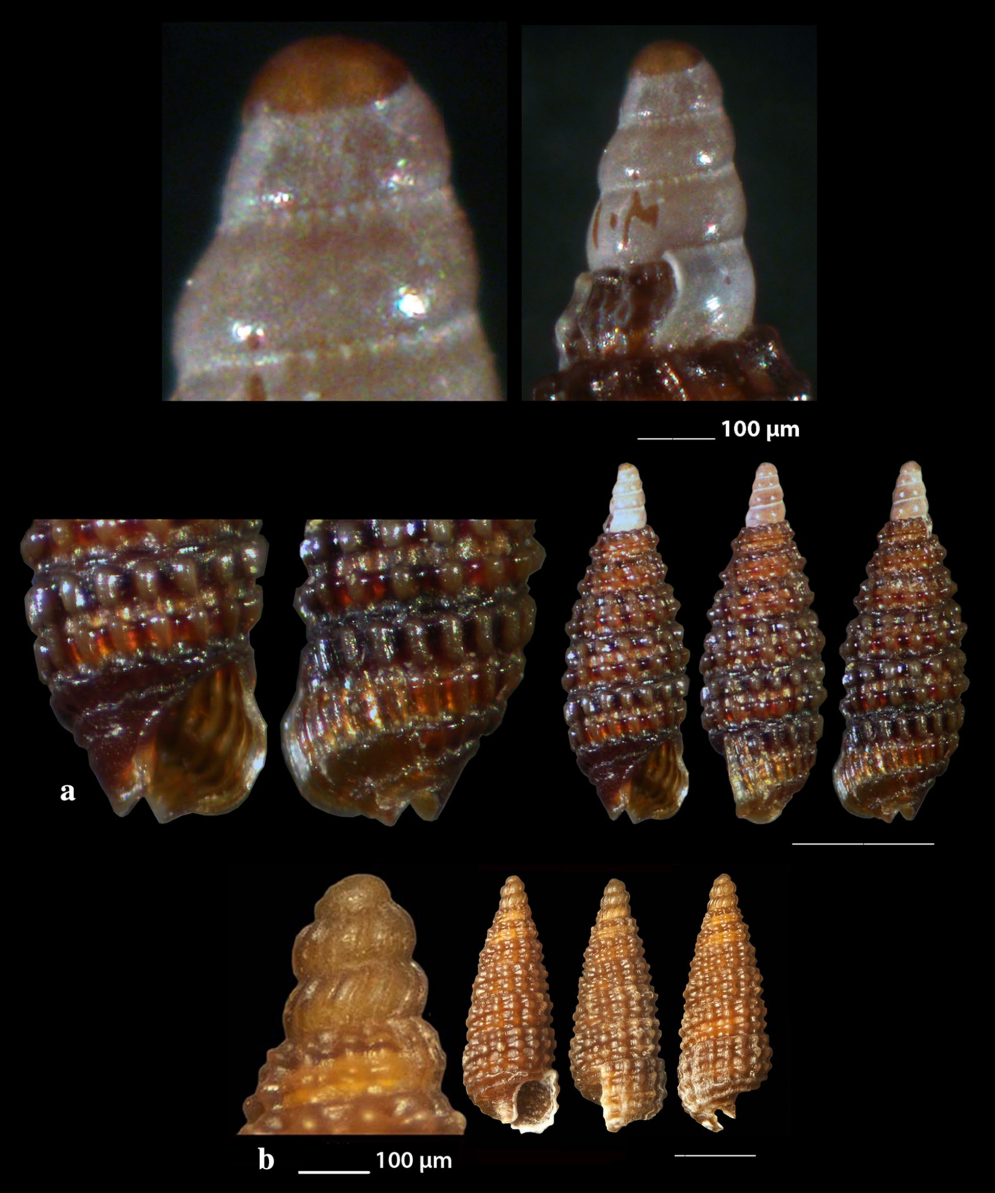
**a** *Horologica* sp., **b** *Krachia tiara*. Bar = 1 mm, unless otherwise indicated

**Fig. 15:**
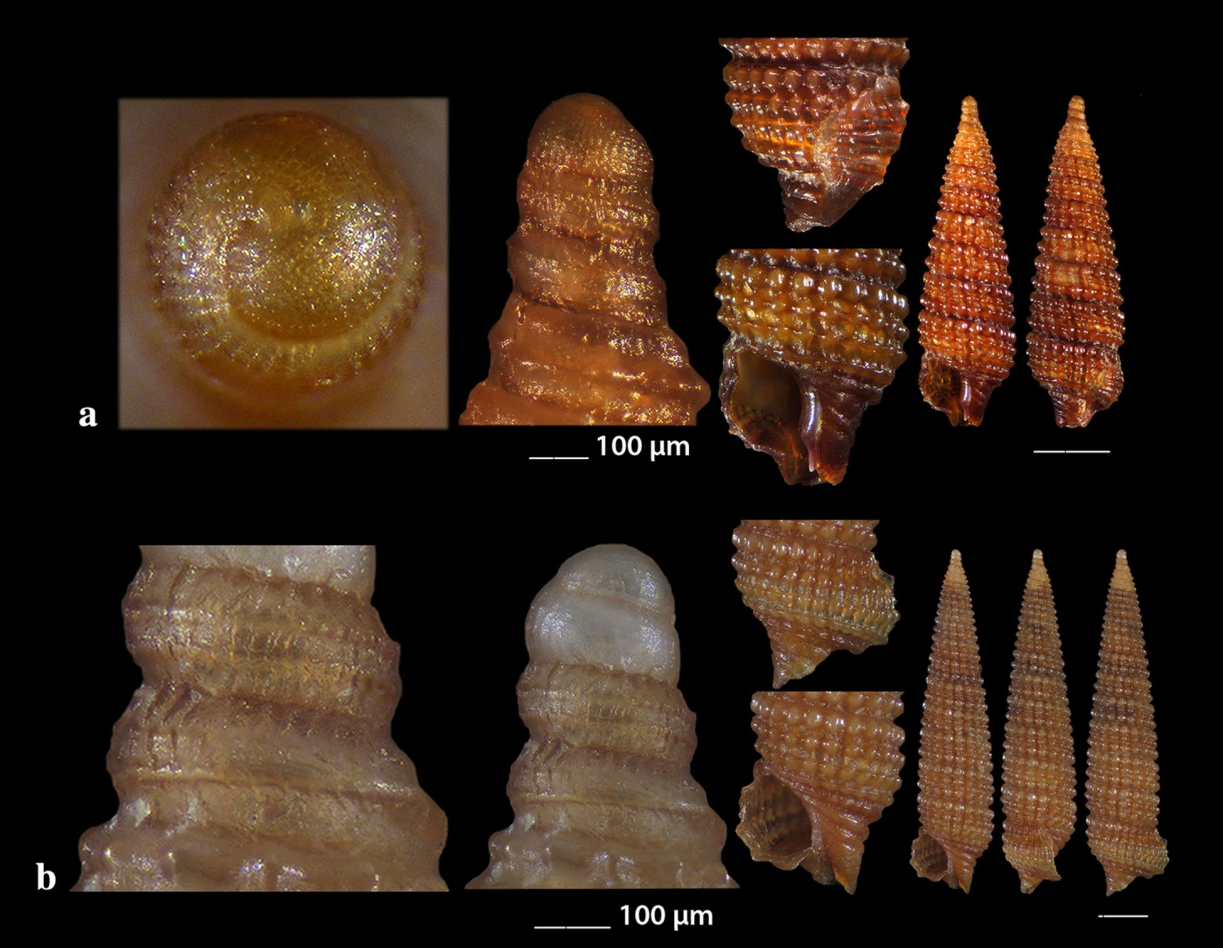
**a**, **b** *Monophorus amicitiae*. Bar = 1 mm, unless otherwise indicated. Specimen 15B courtesy of Panagiotis Ovalis

**Fig. 16:**
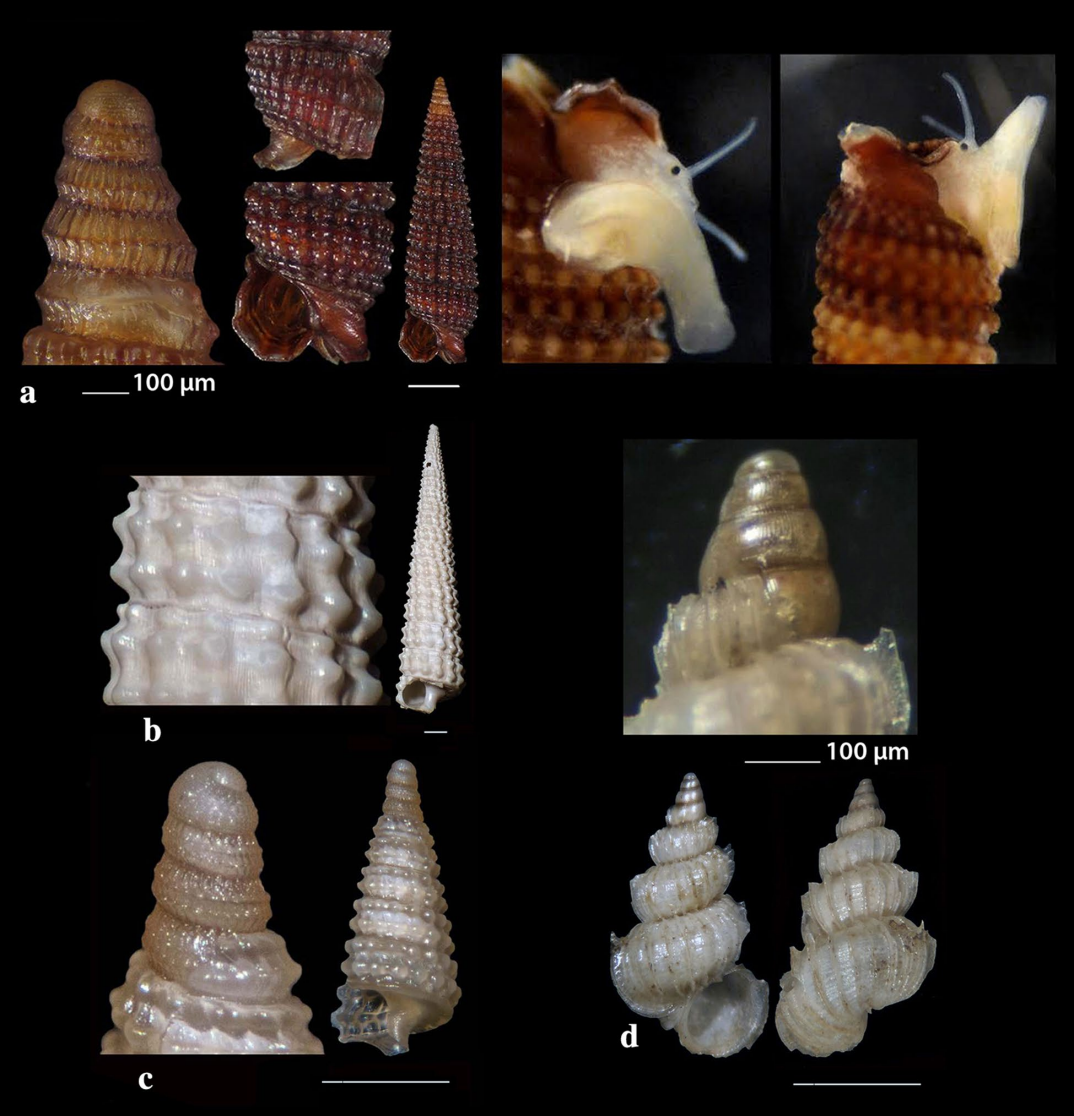
**a** *Obesula marisnostri*, **b**, **c** *Strobiligera brychia*, **d** *Epitonium tryoni*. Bar = 1 mm, unless otherwise indicated

**Fig. 17:**
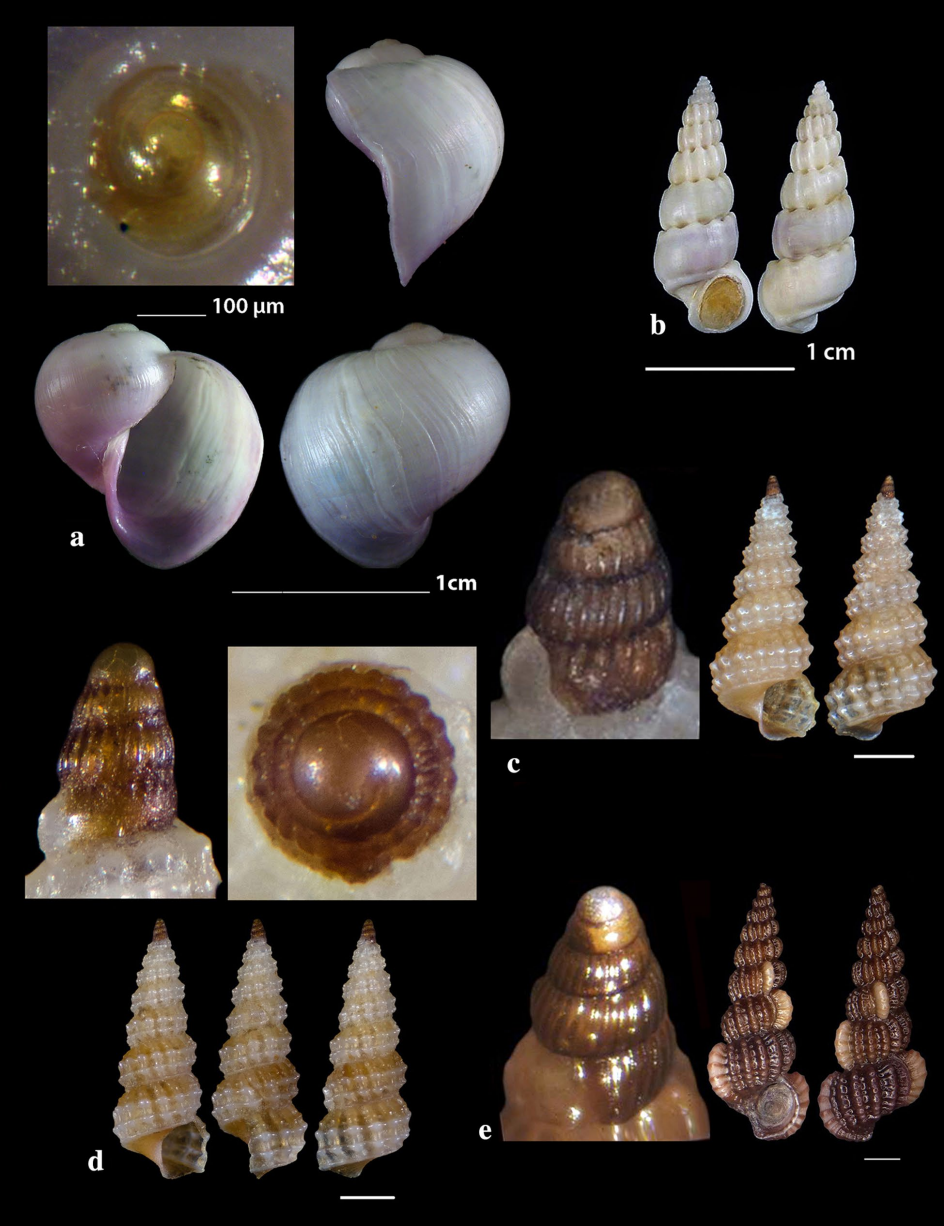
**a** *Janthina pallida*, **b** *Opalia crenata*, **c**, **d** *Narrimania concinna*, **e** *Opaliopsis atlantis.* Bar = 1 mm, unless otherwise indicated

**Fig. 18:**
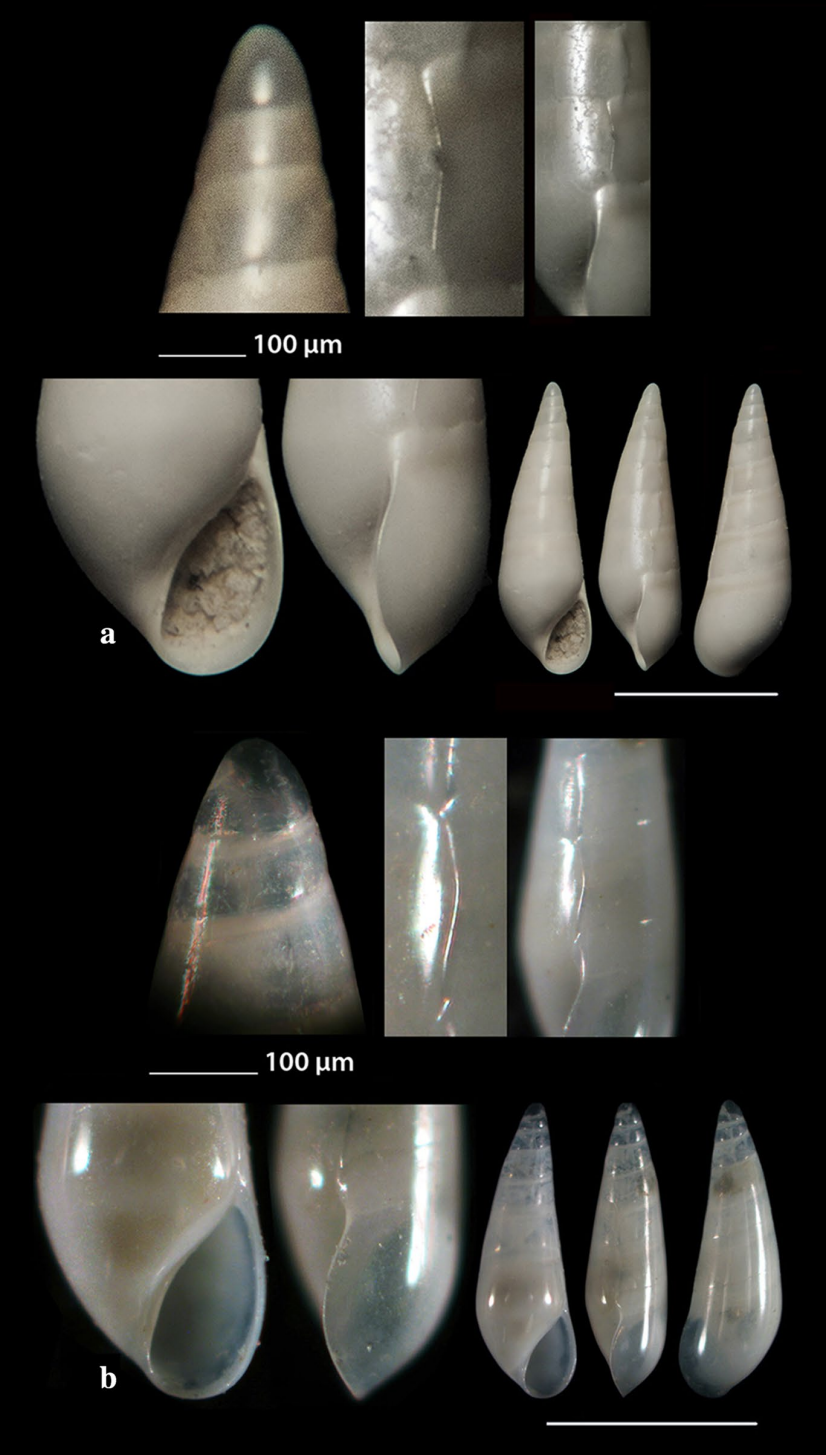
**a**, **b** *Acrochalix* cf. *callosa*. Bar = 1 mm, unless otherwise indicated

**Fig. 19:**
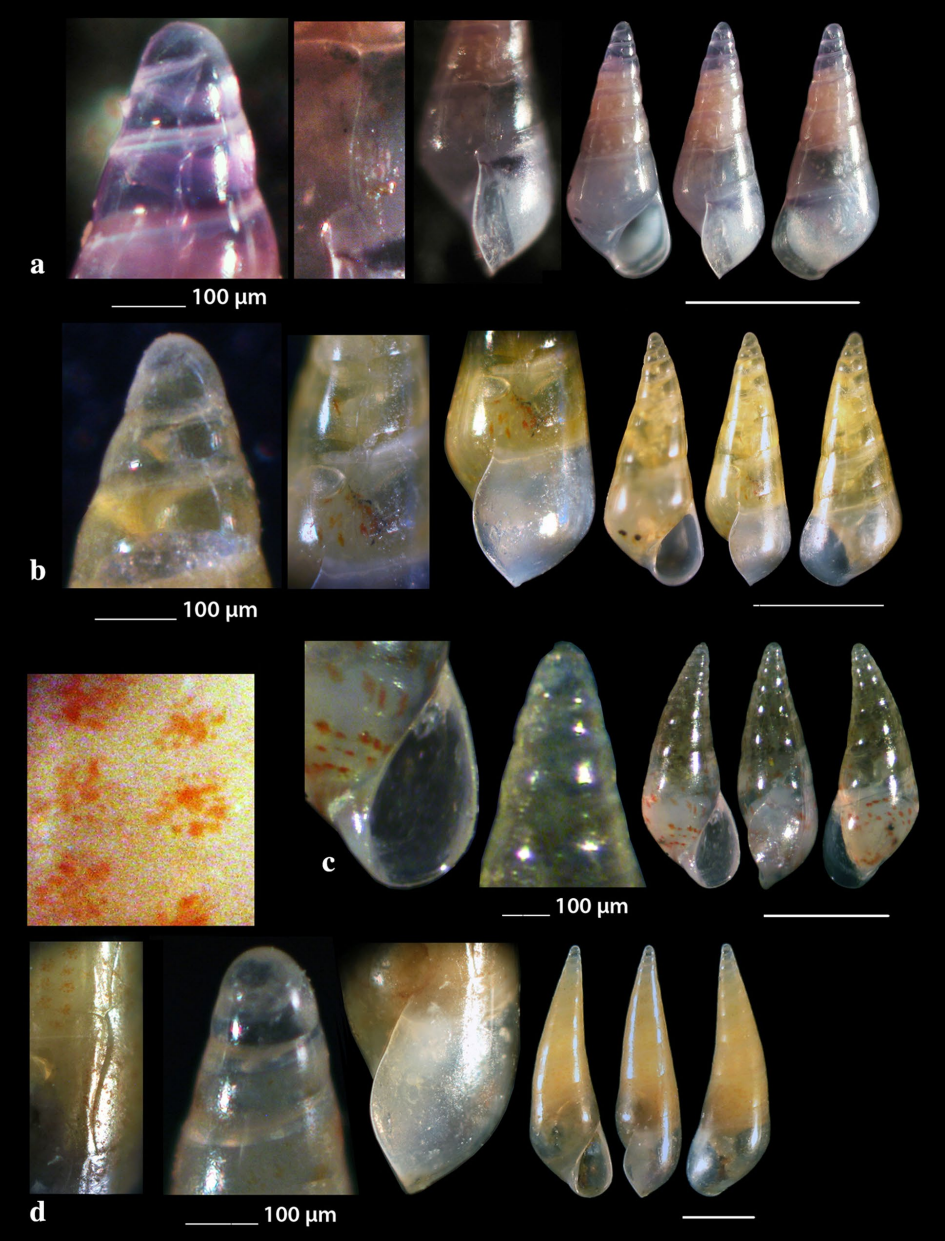
**a** *Campylorhaphion* cf*. famelicum*, **b** *Melanella spiridioni*, **c**, **d** *Curveulima dautzenbergi*. Bar = 1 mm, unless otherwise indicated

**Fig. 20:**
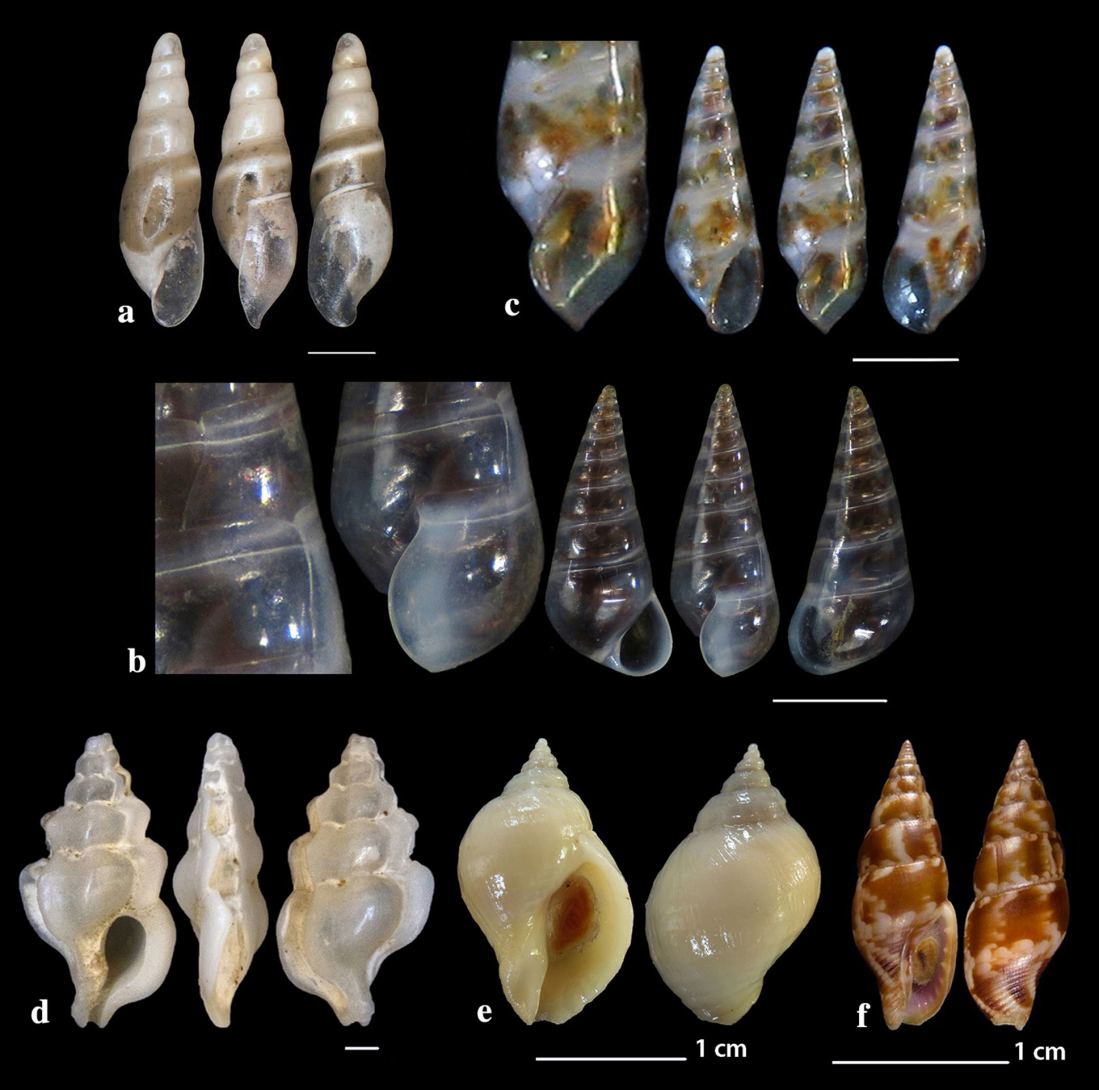
**a** *Haliella stenostoma*, **b** *Nanobalcis nana*, **c** *Sticteulima jeffreysiana*, **d** *Aspella anceps*, **e** *Nucella lapillus*, **f** *Mitrella pallaryi*. Bar = 1 mm, unless otherwise indicated

**Fig. 21:**
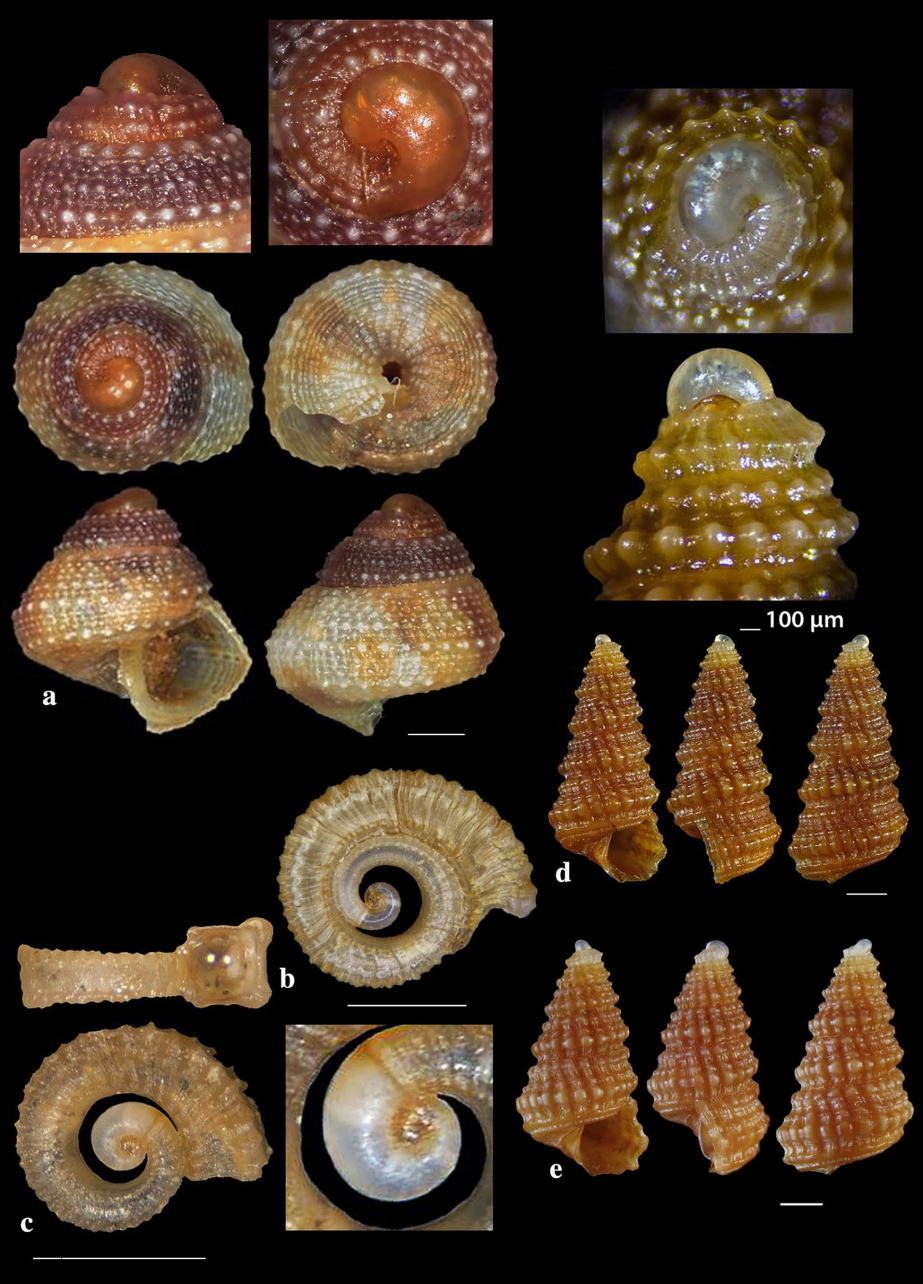
**a** *Heliacus jeffreysianus*, **b**, **c** *Spirolaxis clenchi*, **d**, **e** *Mathilda coronata*. Bar = 1 mm, unless otherwise indicated

### The documented records for the Hellenic Seas

All 1st and 2nd documented records from the Hellenic Seas are presented bellow per family; a brief description is given whenever additional information is supportive to their identification.

### Fissurellidae Fleming, 1822

*Diodora funiculata* (Reeve, 1850) (Fig. 2a). Three live individuals (20.00–29.00 mm long, 13.45–20.00 mm wide, 8.65–13.00 mm high) from mixed bottoms, − 1 to − 2 m, station 14. A Lessepsian migrant reported from the coast of Israel and established there [23]. It is uncommon in the Egyptian Red Sea [24] while the species is distributed in the Arabian Gulf [25].

*Fissurisepta granulosa* Jeffreys, 1883 (Fig. 2b). One shell (2.30 mm long, 1.00 mm wide, 1.20 mm high), − 400 m, coralligenous bottom, station 1. Circalittoral of deep waters of West and Central Mediterranean; rare [26–29].

### Lepetidae Gray, 1850

*Iothia fulva* (O. F. Müller, 1776) (Fig. 2c). Six shells (1.70–6.30 mm long, 1.40–5.60 mm wide, 1.00–4.00 mm high), − 400 m, coralligenous bottom, station 1. Fresh shells of *I. fulva* bear a yellow to light orange periostracum [28]. The species is distributed in the continental shelf from NE Atlantic to the Azores [27, 28, 30, 31]. It is rare in the Mediterranean Sea [27] mainly found in central Tyrrhenian and Adriatic Seas [29] and occasional in SW Albania [32].

*Propilidium exiguum* (W. Thompson, 1844) (Fig. 2d). Seven shells (1.40–2.80 mm long, 1.20–2.30 mm wide, 0.90–1.60 mm high), − 400 m, coralligenous bottom, station 1. European coastal zone to deeper than 2500 m [28, 33–37].

### Anatomidae McLean, 1989

*Anatoma tenuisculpta* (Seguenza, 1880) (Fig. 3a). Two shells (0.90 and 2.40 mm high, 1.20 and 3.00 mm wide, respectively) − 400 m, coralligenous bottom, station 15. An uncommon species with wide depth distribution range (50 to  > 1800 m) in N and W Europe and in the Mediterranean Sea [38]; numerous shells were found in the Alboran Sea [31].

### Addisoniidae Dall, 1882

*Addisonia excentrica* (Tiberi, 1855) (Fig. 3b). One shell (12.40 mm long, 10.35 mm wide, 5.60 mm high), 50 m, mixed bottom, station 30. Uncommon, West Mediterranean [35, 39, 40].

### Skeneidae Clark W., 1851

*Akritogyra conspicua* (Monterosato, 1880) (Fig. 3c). 23 shells (1.10–1.90 mm high, 1.40–2.40 mm wide), − 400 m, coralligenous bottom, station 1. Uncommon, West Mediterranean [27, 41] and East Mediterranean [42] up to the Sea of Marmara, in depths > 1000 m [43].

*Cirsonella romettensis* (Granata-Grillo, 1877) (Fig. 4a, b). Three shells (1.60–1.80 mm high, 1.60–1.80 mm wide), − 400 m, coralligenous bottom, station 1. Rather common, West Mediterranean [27, 35, 41].

*Lissomphalia bithynoides* (Monterosato, 1880) (Fig. 4c). One shell (0.90 mm high, 1.00 mm wide), mixed bottom, − 80 m, station 27. Common, Mediterranean, of infralittoral to bathyal distribution [27, 36, 41, 44].

### Chilodontidae Wenz, 1938

*Danilia costellata* (O.G. Costa, 1861) (Fig. 5a–c). One live specimen and 13 shells (2.65–10.00 mm high, 2.55–6.55 mm wide), biogenic material and maerl, 80–120 m, stations 23, 24 and 25. Protoconch paucispiral of one smooth whorl followed by half a rather smooth whorl with axial ribs. Next whorls are decorated with distinct wavy spiral cords. The body whorl bears 14–15 axial prosocline (15^o^–20^o^) ribs crossed by spiral cords and forming shallow cavities. Columellar tooth variable from small to big in size and from pointed to square in form. 14–15 main teeth on the outer lip and several secondary. Unicolor of moccasin color in general. Very similar to *D. tinei* (Calcara, 1839) (Fig. 5d) but the latter bears 17–18 axial prosocline cords (30^o^–35^o^) while the cavities of the decussated areas are deeper. Its outer lip bears less main teeth (10–12) and the shell is whitish with about 8 brown zigzag zones of flames (not as spots) [35]. *Danilia costellata* was referred from the Mediterranean Sea by Palazzi and Villari [45], Crocetta and Spanu [46] and Cossignani et al. [47] as certain, by Vilvens and Heros [48] as doubtful and Herbert [49] characterized that of Crocetta and Spanu [46] as tentative. In contrast, *D. tinei* was referred from the Mediterranean Sea by many more researchers including Gofas et al. [35], Ghisotti and Steinmann [50], Cachia et al. [51], Navaro and Capdevila [52], Scaperrotta et al. [53] and Öztürk et al. [54]. Based on the available bibliography, the two Mediterranean *Danilia* species have overlapping taxonomical characteristics including the shell outline, the ratio height/width, the shape and size of the collumellar tooth and the depth distribution range. Moreover, as the juvenile shells exhibit no differences [55], a further study of these two congeneric species seems to be necessary.

### Trochidae Rafinesque, 1815

*Gibbula* cf. *vimontiae* Monterosato, 1884 (Fig. 6a). One live juvenile specimen (0.85 mm high, 0.95 mm wide), − 40 m, mixed bottom, station 26. It is presented as *G.* cf. *vimontiae* due to its more globular outline and the smaller size than that presented by Scaperotta et al. [59]. *Gibbula vimontiae* is uncommon in the West Mediterranean [27], quite common in beached detritus in Tunisia, or Mediterranean [56, 57], North Atlantic [58] and Rhodes [59].

### Neritidae Rafinesque, 1815

*Smaragdia souverbiana* (Montrouzier in Souverbie & Montrousier, 1863) (Fig. 6b, c). One live individual and two shells (2.32–3.30 mm high, 2.50–3.35 mm wide) mixed bottom, 0–40 m, stations 14, 17 and 23. *Smaragdia souverbiana* is a known Lessepsian reported from Cyprus and Turkey [6] and recently from Lesvos Island, NE Aegean Sea [60, 61]. In Australia, this species consumes various seagrass species with a strong preference for *Zostera capricorni* Ascherson, 1876 and *Halophila ovalis* (R. Brown) J.D. Hooker, 1858 [62]. The Lessepsian angiosperm *Halophila stipulacea* (Forsskål) Ascherson, 1867 was referred from all places where *S. souverbiana* was recorded, assuming that *H*. *stipulacea* might also be the food of *S. souverbiana* in the Mediterranean Sea.

### Cerithiidae Fleming, 1822

*Rhinoclavis kochi* (Philippi, 1848) (Fig. 7a–c). 32 shells (5.30–36.50 mm high, 2.00–9.50 mm wide), sandy bottom, − 1 to − 3 m, stations 19 and 20. The species was characterized as one of the 100 “Worst Invasive” [63].

### Newtoniellidae Korobkov, 1955

*Cerithiella metula* (Lovén, 1846) (Fig. 7d). Two shells (2.50 and 3.80 mm high, 0.90 and 1.40 mm wide, respectively), − 400 m, coralligenous bottom, station 1. It is mainly distributed in NE Atlantic, being extremely rare in the Mediterranean Sea [64] or rare in Central and East Mediterranean [27]. Gofas et al. [31] do not confirm the presence of the species in the Mediterranean Sea.

### Cerithiopsidae H. Adams & A. Adams, 1853

*Cerithiopsis annae* Cecalupo & Buzzurro, 2005 (Fig. 8a). One live specimen (3.40 mm high, 1.35 mm wide), in *Aplysina aerophoba* (Nardo, 1833) sponge host, − 8 m, station 6. The species is compared with *C. tubercularis* (Montagu, 1803), *C. nana* Jeffreys, 1867 and *C*. *minima* (Brusina, 1865) (see Figs. 8b, 9a, b, respectively). It is recorded in shallow waters of the West [47, 65, 66] and Central Mediterranean Sea [57, 67].

*Cerithiopsis buzzurroi* (Cecalupo & Robba, 2010) (Fig. 10a, b). Three live specimens and nine shells (2.65–4.45 mm high, 0.95–1.45 mm wide), mixed bottom, − 40 m, station 5; − 6 to − 8 m, station 20; − 50 to − 90 m, mixed bottom, station 23. Sublittoral, Mediterranean [67].

*Cerithiopsis denticulata* (Cecalupo & Robba, 2010) (Fig. 11a–d). Five shells (2.35–5.35 mm high, 0.85–1.35 mm wide), − 7 m, in *Aplysina aerophoba* (Nardo, 1833) sponge host, station 2; − 15 m, *Spongia (Spongia) officinalis* Linnaeus, 1759 sponge host, station 5; *Zostera* bed, − 0.2 m, station 6; − 70 m, hard substrate, small scale fishing nets, station 23. Infralittoral and upper circalittoral throughout the Mediterranean Sea [67].

*Cerithiopsis jeffreysi* Watson, 1885 (Fig. 11e, f). Two live specimens (1.90 and 3.10 mm high, 0.75 and 1.00 mm wide, respectively), − 3 and − 8 m, in *Aplysina aerophoba* (Nardo, 1833) sponge hosts, stations 6 and 25. Uncommon [68], Mediterranean [27, 35, 44]. It is also recorded from NE Turkish Aegean Coasts [54].

*Cerithiopsis ladae* Prkić & Buzzurro, 2007 (Fig. 12a). Five live specimens and two shells (1.60–2.00 mm high, 0.75–0.90 mm wide), in *Aplysina aerophoba* (Nardo, 1833) sponge host, − 4 and − 8 m, stations 3 and 6; *Cladocora caespitosa* (Linnaeus, 1767) scleractinian, − 40 m, station 5. Uncommon, West and Central Mediterranean Sea [29, 35, 69–71].

*Cerithiopsis oculisfictis* Prkić & Mariottini, 2010 (Fig. 12b). Two live specimens (3.80 and 4.00 mm high, 1.00 and 1.10 mm wide, respectively), − 10 m, among *Cladocora caespitosa* (Linnaeus, 1767) scleractinian coral branches, station 5. Adriatic Sea [72].

*Cerithiopsis petanii* Prkić & Mariottini, 2010 (Fig. 13a). One live specimen (6.70 mm high, 1.65 mm wide), − 10 m, mixed bottom, station 5. Known from intertidal zone of the Adriatic Sea in sponges [72].

*Cerithiopsis pulvis* (Issel, 1869) (Fig. 13b). Two shells both with chipped off protoconches (2.70 and 3.85 mm high, 1.00 and 1.30 mm wide, respectively), − 10 m, mixed bottom, stations 20 and 21. A Lessepsian migrant in the East Mediterranean, expanded from the Israel coasts and East Aegean Sea northern up to the Black Sea [73, 74].

*Cerithiopsis scalaris* Locard, 1891 (Fig. 13c). One live specimen and nine shells (3.00–5.45 mm high, 1.00–1.90 mm wide), − 10 m, station 5; muddy bottom, − 60 m, station 9; − 70 m, rocky bottom, station 23. Uncommon, infralitoral rocky zone, associated with sponges, Mediterranean Sea [27, 35, 75, 76].

Another specimen present as *C*. cf. *scalaris* Locard, 1891 (Fig. 13d, not in Table 1) was collected, mixed bottom, − 10 m, station 5. It is lighter in color (honey-yellow) with the adapical cord light brown instead of unicolor and dark chestnut-brown as referred by Gofas et al. [35], and with a cylindroconical protoconch of 4.5 whorls instead of 3.5–4.0 of *C. scalaris* [77, 78]. Oliver et al. [79] pointed out that the teleoconch of *C. scalaris* may vary from cylindrical to cyrtocylindrical, that members of the genus *Cerithiopsis* with the same type of protoconch differ in their teleoconch and that cryptic species possibly exist within the *C. scalaris* complex.

*Horologica* sp. (Fig. 14a). One live specimen (2.55 mm high, 0.90 mm wide), *Aplysina aerophoba* (Nardo, 1833) sponge host, − 6 m, rocky bottom, station 16. Shell small, h/w ratio 2.8, fusiform, glossy, slightly scalaroid and moderately slender. Protoconch 400 μm high and 220 μm wide, conical, smooth, milky-white with light brown nucleus, translucent, consisting of about 4 slightly convex whorls less inflated adapically and exhibiting minute and dense dentition at the suture. Limit to the teleoconch sigmoidal. Teleoconch composed of 5.5 flat whorls with a broad base. Spiral sculpture of two well-separated cords starting as two from the onset of the teleoconch, the adapical one being weaker in the first three whorls but becoming first equal and finally stronger in the last whorl. Two additional wide and strongly tuberculated spiral cords—peripheral and basal—and a swelling before the lower end. Axial sculpture of 14 strong, prosocline and axially aligned ribs on the body whorl, which at their intersections with the spiral cords form ovate, equidistant, conspicuous nodules with quadrangular interspaces. The nodules are elongated radially in the first four whorls and axially in the last one. Suture deep and evident separating clearly the whorls. Color walnut-brown with lighter nodules and the two basal cords darker brown. Aperture pear-shaped, wide, with white and simple outer lip. Columellar callus conspicuous, peristome thin, anal canal short and broad, siphonal canal short, open and stubby. Aperture showing by transparency the pattern of the spire. Anterior part of the animal grayish-white. The shell looks similar to *Cerithiopsis minima* (Brusina, 1865) in its outlook, the size and the white planktotrophic larval shell (only lacking the minute denticles in *C*. *minima*), but it bears two spiral cords as in the genus *Dizoniopsis* Sacco, 1895 and *Horologica* Laseron, 1956 [80]. It also looks similar to *Dizoniopsis aspicienda* Bouchet, Gofas & Warén, 2010, but it differs mainly in the type of protoconch and the color. As members of the genus *Dizoniopsis* bear a stiliform or globose larval shell decorated with axial ribs or spiral cords the specimen most probably belongs to the genus *Horologica* Laseron, 1956, the members of which bear a smooth multispiral conical/cylindrical protoconch and 2 spiral cords per whorl [80, 81]. Oliver et al. [82] under the name *Dizoniopsis* sp. (Figure 77 in [82]) show a quite similar to our shell, with missing protoconch, from the Chafarinas Islands, Alboran Sea. The latter authors refer that their specimen differs from the known *Dizoniopsis* species from the Atlantic Ocean and the Mediterranean Sea.

*Krachia tiara* (Monterosato 1874) **(**Fig. 14b). One shell (3.50 mm high, 1.30 mm wide), mixed bottom, − 80 m, station 27. An uncommon Mediterranean species [27, 35, 47, 74] was also recorded in East Mediterranean Sea from the Turkish coasts [54].

### Triphoridae Gray, 1847

*Monophorus amicitiae* Romani, 2015 (Fig. 15a). One shell (4.30 mm high, 1.20 mm wide) from maerl beds, − 70 to − 90 m, station 23; two additional shells (Fig. 15b) (7.15 and 7.40 mm high, 1.60 and 1.65 mm wide, respectively) were collected from the same station and habitat and were kindly offered by Panagiotis Ovalis to be included in this publication. Known from the infralittoral zone of the Northern Tyrrhenian Sea [83, 84].

*Obesula marisnostri* Bouchet, 1985 (Fig. 16a). One live mature specimen of dark chestnut-red shell (7.55 mm high, 1.90 mm wide) and lighter apex, in whitish sponge on maerl, − 100 m, station 23. The easily recognizable shell has been well described [21, 35, 85] but the animal coloration was lacking until now where one live specimen has been collected. The external parts are uniformly translucent-white with cream-white spots and black eyes. The species has been referred from Greece [21] and other areas of the Mediterranean Sea [35, 77].

*Strobiligera brychia* (Bouchet & Guillemot, 1978) (Fig. 16b, c). Two shells (2.20 and 14.00 high, 0.95 and 3.35 mm wide, respectively), − 400 m, coralligenous bottom, station 1. On rocky circalitoral and bathyal bottoms from the East Atlantic to the West Mediterranean Sea [27, 28, 35, 77].

### Epitoniidae Berry, 1910 (1812)

*Epitonium tryoni* (de Boury, 1913) (Fig. 16d). One shell (2.30 mm high, 1.10 mm wide), trawled, rocky bottom, − 200 m, station 30. Uncommon, NE Atlantic and West Mediterranean Sea [27, 36, 74, 86].

*Janthina pallida* W. Thompson, 1840 (Fig. 17a). Several shells (12.90–17.00 mm high, 11.95–15.75 mm wide), beached, station 28. A cosmopolitan species frequently beached in the Mediterranean Sea [27, 35, 36] but rare in the Maltese waters [44].

*Opalia crenata* (Linnaeus, 1758) (Fig. 17b). One live specimen and six shells (10.50–17.00 m high, 4.15–6.69 mm wide), beached, 5–10 m of mixed bottom, station 29. It is an uncommon amphiatlantic and Mediterranean species of shallow waters [27, 35, 36] associated with *Anemonia sulcata* [35].

### Nystiellidae Clench & Turner, 1952

*Narrimania concinna* (Sykes, 1925) (Fig. 17c, d). One live specimen and one shell (4.10 and 4.90 mm high, 1.55 and 1.80 mm wide, respectively) − 135 m, station 10; − 90 m, hard substrate, station 23. Rare, South and Central Mediterranean and Atlantic [27, 86, 87].

*Opaliopsis atlantis* (Clench & Turner, 1952) (Fig. 17e). One live specimen and five shells (4.00–9.00 mm high, 1.20–3.50 mm wide), − 200 m, rocky bottom, station 1 and − 400 m, coralligenous bottom, station 2. Very rare in the Mediterranean Sea, less rare in Cuba, Florida and the Azores [27, 35, 44, 86, 88–90].

### Eulimidae Philippi, 1853

*Acrochalix* cf. *callosa* Bouchet & Warén, 1986 (Fig. 18a, b). Two shells (1.40 and 1.85 mm high, 0.50 and 0.60 mm wide, respectively), − 450 m, station 1; − 70 m, station 10. The solid, pointed and gently curved shell bears a conical protoconch. The later has a bluntly rounded initial whorl, a visible limit with the teleoconch, consists of 2.5 evenly convex whorls, is 260 μm high and 185 μm wide. The teleoconch bears 6 perfectly smooth and nearly flat whorls of gradually increasing diameter and an evenly curved axis. Suture very indistinct while the incremental growth scars of all 6 whorls are strictly aligned, forming thus a perfect series oriented towards the upper right of the shell. The right side of the shell is almost straight. The shell is flattened dorso-ventrally with the diameter from the outer lip to the opposite side of the shell measuring 578 μm and that at a right angle to this measuring 522 μm. The relation is 0.90. The last whorl occupies nearly 50% of the shell’s length. Aperture high and pyriform with its long axis towards the right, outer lip orthocline joining the suture at a right angle but slightly bending to the right at its high most end and slightly projecting at its lower part and at 2/5 of its height. Inner lip reflected both on the columella and the parietal wall forming a continuous callus while the columella is straight in its upper part, is curved in its lower part and continuous with the parietal wall. Outer lip sinuous at its vicinity with the suture. *Acrochalix callosa* is reported as a NE Atlantic species and differs from other Mediterranean minute and curved species by having a slender aperture, a straighter columella–parietal wall line and a well-developed, solid and continuous inner lip [86]. As the species is very rare and its variability unknown, we refer to our specimens as *A*. cf. *callosa*.

*Campylorhaphion* cf*. famelicum* (Watson, 1883) (Fig. 19a). One young live specimen (1.50 mm high, 0.60 mm wide), on *Holothuria (Holothuria) tubulosa* Gmelin, 1791 host, − 35 m, mixed bottom, station 4. The hyaline, white, club-shaped shell has a paucispiral protoconch of 2 whorls (excluding the nucleus) and a diameter of approximately 200 μm. The suture of the protoconch whorls exhibits a weak and dense crenulation. The teleoconch is slightly curved, of 5 shiny whorls that are nearly smooth though with very fine axial sculpture approximately 30 μm apart that is more prominent close to the incremental scars (Fig. 19a). The body whorl occupies 48% of the shell’s length and forms a rounded base, while the aperture is nearly twice as high as wide and occupies 34% of the shell’s length. Its *Vitreolina* type incremental scars are not strictly aligned, are facing the lateral side of the shell over the outer lip with the two last scars (above the outer lip) in a slowly retreating series, nearly aligned with the outer lip and the two adapical (older ones) in advancing series, while the oldest one is more conspicuous. The inner lip is straight by the columella and projected over its lower part, while the outer lip is arquated and orthocline. Animal of light pink-purple color. We did not manage to find in the literature a description of a specimen of the same developmental stage as ours, therefore, we present the later as *C.* cf. *famelicum*. The specimen resembles *Melanella spiridioni* (Dautzenberg & H. Fisher, 1896) (Fig. 19b) from which it differs in having a cylindrical protoconch instead of a conical of *M*. *spiridioni* and a more fragile teleoconch, in its aperture which is, at least, twice as high as wide and in the color of the animal which is lemon-yellow with red speckles in *M*. *spiridioni*. It differs from the *Vitreolina* species in having a cylindrical protoconch with almost 2½ whorls. *Campylorhaphion famelicum* is uncommon in the Central Mediterranean Sea [27] and in the North Atlantic [86].

*Curveulima dautzenbergi* (Pallary, 1900) (Fig. 19c, d). Four live specimens and two shells (1.50–3.50 mm high, 0.60–1.15 mm wide), detritus material, − 40 m, mixed bottom, station 3. Uncommon, in subtidal rocky bottoms parasitizing crinoids of the genus *Antedon* in the Atlantic and the West Mediterranean Sea [35, 91].

*Haliella stenostoma* (Jeffreys, 1858) (Fig. 20a). One shell (4.30 mm high, 1.30 mm wide), − 400 m, coralligenous bottom, station 1. A rare North Atlantic and Mediterranean species of muddy circalittoral and bathyal planes [27, 86, 92, 93].

*Nanobalcis nana* (Monterosato, 1878) (Fig. 20b). 12 live individuals and several shells (2.00–2.45 mm high, 0.90–1.10 mm wide), mixed bottom, − 40 to − 120 m, station 13; − 40 to − 70 m, kelps, station 23. Up to 2001, the species was recorded only in the Central Mediterranean [94] but eventually was found by Öztürk et al. [54] in the Turkish coast of the SE Aegean Sea. It is characterized as uncommon [27, 35] in the Mediterranean Sea and is a parasite of sea urchins [35, 94].

*Sticteulima jeffreysiana* (Brusina, 1869) (Fig. 20c). Seven live specimens and 10 shells (2.25–2.70 mm high, 0.70–0.95 mm wide), from mixed bottoms − 70 m, station 6; − 40 m, station 10; − 30 m, station 11; − 30 m, station 12; − 60 m, station 13; − 40 to − 50 m, mixed bottom with kelps, station 23, were all collected from detritus material. The species is distributed all over the Mediterranean Sea [27, 35, 44] and from the Hellenic Seas it was referred from the South Aegean Sea [16].

### Muricidae Rafinesque, 1815

*Aspella anceps* (Lamarck, 1822) (Fig. 20d). Six shells (7.00–11.00 mm high, 3.50–5.00 mm wide), − 6 to − 10 m, mixed bottom, station 21. A rare and characteristic inhabitant of the East Mediterranean [27].

*Nucella lapillus* (Linnaeus, 1758) (Fig. 20e). Two live specimens (18.65 and 25.70 mm high, 11.60 and 15.70 mm wide, respectively), detritus material, − 10 m, mixed bottom, station 8. Uncommon, carnivorous, West and Central Mediterranean [27, 35, 47].

### Columbellidae Swainson, 1840

*Mitrella pallaryi* (Dautzenberg, 1927) (Fig. 20f). Four live specimens and three shells (15.00–18.00 mm high, 5–6 mm wide), biogenic substrate, − 30 to − 70 m, station 23. Uncommon, Mediterranean, coralligenous, sandy and muddy circalittoral and bathyal bottoms from 30 to 250 m [27, 35].

### Architectonicidae Gray, 1850

*Heliacus jeffreysianus* (Tiberi, 1867) (Fig. 21a). One live specimen (3.75 mm high, 3.85 mm wide), − 100 m, maerl, station 16. *H. jeffreysianus* is an extremely rare architectonicid in the Mediterranean Sea and worldwide [95–97].

*Spirolaxis clenchi* Jaume & Borro, 1946 (Fig. 21b, c). Two shells (0.50 and 0.75 mm high, 1.50 and 2.30 mm wide, respectively), coralligenous bottom, − 400 m, station 1. Rare species of the deep and bathyal Atlantic and Mediterranean [27, 35, 98].

### Mathildidae Dall, 1889

*Mathilda coronata* Monterosato, 1875 (Fig. 21d, e). Two shells (4.95 and 6.35 mm high, 2.75 and 2.95 mm wide, respectively), − 80 and − 120 m, biogenic substrate and maerl, stations 16 and 31. Rare, circalittoral and bathyal, Mediterranean [27, 35].

## Discussion

**Fig. 22:**
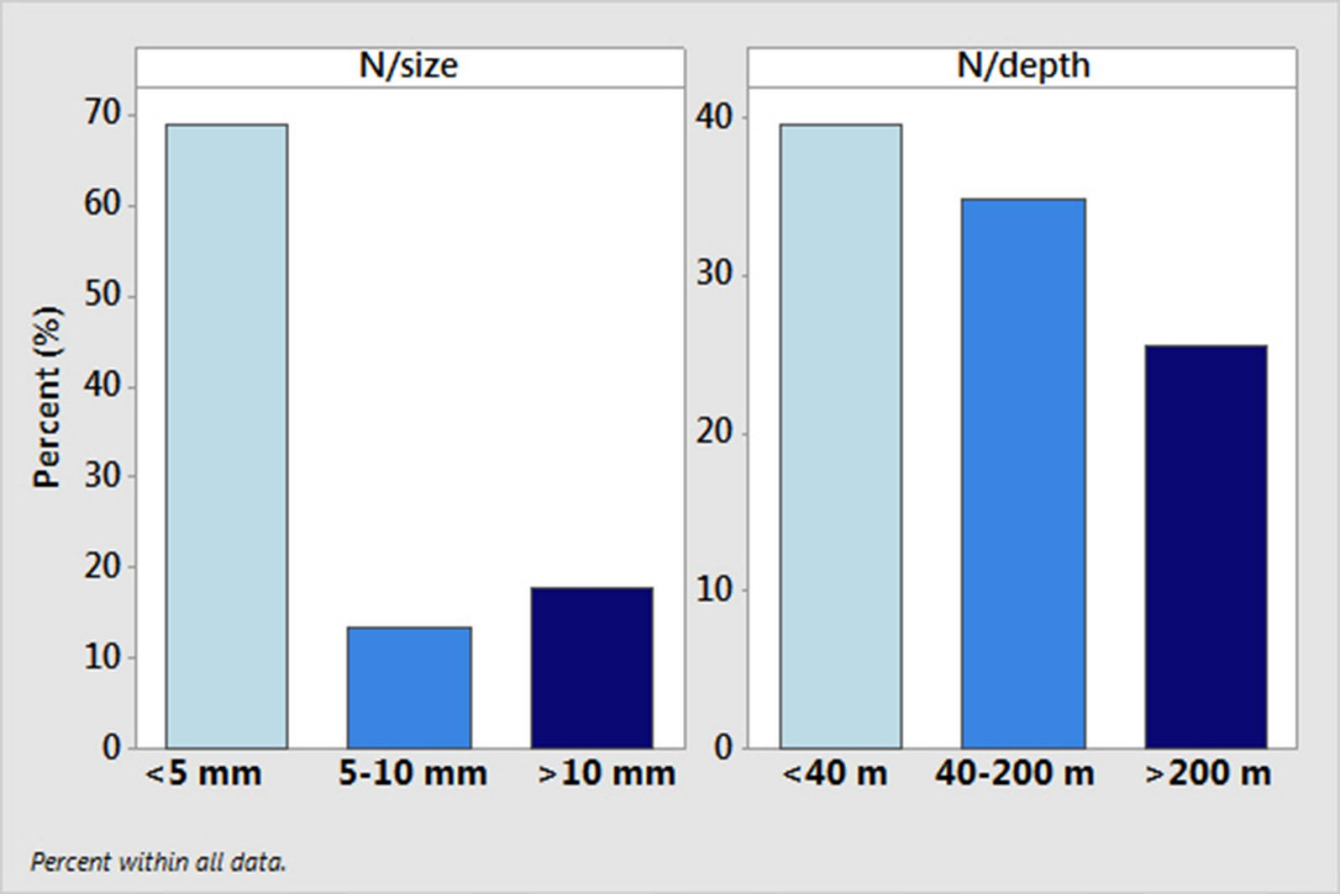
Percentage of species number (N) per size and depths of collection

Out of the 45 presented species, 40 are reported for the first time for the Hellenic fauna, raising its gastropod biodiversity from 651 [21] to 691 species, and 17 species are new records for the East Mediterranean Sea. Two families and 19 genera are also new records for the Hellenic fauna (see Table 1). Interestingly, the vast majority of the presented species is of minute size (nearly 70%) being collected from a variety of substrates and depths (Fig. 22).

The occurrence of only shells and among them juvenile ones of *Rhinoclavis kochi* accumulated in shallow water stations, could be explained by an initial rapid increase of the population density followed by a decline due to the sea temperature change [5] and subsequent accumulation of the shells by the sea currents. This may also mean a temporal establishment. A future occurrence of live specimens could not be excluded provided the environmental conditions are favorable and the corridors are open.

The almost simultaneous findings of *Cerithiopsis buzzurroi*, *C. ladae*, *C. scalaris*, *Narrimania concinna*, *Nanobalcis nana* and *Sticteulima jeffreysiana* from different areas of Greece indicates that they are more widely distributed in the Hellenic Seas and the Eastern Mediterranean Sea, and that targeted researches from the same type of substrates and direct sampling might bring to light more species.

Field observations and the gross anatomy of their alimentary systems indicate that ptenoglossans are parasites [99]. Among them, cerithiopsids are usually associated with sponges while eulimids have a strict preference for certain classes of echinoderms [99]. At the same time the identification of cerithiopsids, in particular, necessitates the presence of intact protoconches and/or the observation of the color of the living animal and thus, the collection of live specimens. In the present study we have met those prerequisites by means of brushing their hosts and by keeping the animals alive until they were photographed.

After an extended survey of the literature on Eritrean and Indo-Pacific Triphorids and the lack, up to now, of intermediate records of *M. amicitiae* from the East Mediterranean Sea [83] almost rules out an Eritrean or Indo-Pacific origin of the species. The present new records from Greece, although cannot challenge that hypothesis, are indicative of a wider range of distribution.

The assignment of our specimen *Horologica* sp. to the genus *Horologica* Lasero, 1956 was based primarily upon the combination of its smooth protoconch (characteristic for the genera *Cerithiopsis* sp., *Horologica* sp. and *Joculator* sp.) and its spiral sculpture of 2 cords (genera *Dizoniopsis sp.*, *Mentax* sp. and *Prolixodens sp.*). *Cerithiopsis* sp. and *Joculator* sp. were excluded as they bear 3 spiral cords while *Dizoniopsis* sp., *Mentax* sp. and *Prolixodens* sp. were excluded on the bases of their ribbed protoconches [80]. We hope that further and persistent efforts will bring to light more specimens to study the variability of the species and perhaps describing a new taxon.

The occurrence of *Heliacus jeffreysianus* in the Hellenic waters, besides the record from South Crete [97] considerably enlarges its distribution, which, apart from its extreme rarity, is indicative of a rather broad range of occurrence. The large hyperstrophic protoconch of about 1 mm, fitting the size range of those species with a planktotrophic development, supports the hypothesis of a potentially wide geographical distribution [100]. To our knowledge, the scattered occurrence and extreme rarity of *H. jeffreysianus* may be attributed to its cryptic life-style which may be intimately linked to a deep water zoanthid host, typical of some offshore bottoms [96].

## Conclusions

45 species, the majority of which is of minute size, belonging to 19 families were identified. Among those species, 17 are new for the Eastern Mediterranean Sea and 40 are new for the Hellenic fauna. The high number of new findings is attributed to the sampling methods applied, the under- or unsearched marine environments investigated, as well as the different types of substrates and depths covered, and the multilateral co-operation.

## Methods

The sampling and handling of the specimens were conducted according to Manousis and Galinou-Mitsoudi [21] from October 2008 to March 2017 in certain locations throughout the Hellenic Seas (Fig. 1). In addition, we have applied: (a) brushing with a soft brush on holothurians, sea urchins and sponges either in situ or on live material brought temporarily to the surface in several localities and from various habitats, (b) sampling vertical substrates (e.g. poles, embankments) by means of a metal pole scraper connected to a 120 μm mesh nylon net, (c) keeping until examined and sorting out benthic material in cool sea water until photography of live specimens took place. Cooling of biological material in the sea water was achieved by means of a gel freeze ice pack under the examination vessel.

The protoconch whorls were counted according to Verduin [101], the protoconch’s visible height was measured parallel to its axis, from the tip to the intersection of the larval scar and the teleoconch suture, while for the shell’s slenderness (h/w) the outer lip of the aperture was included in the shell’s width.

The species recognition was based on: (a) systematic guides and atlases (e.g. [5, 27, 29, 35]); (b) faunistic and review articles [76, 102], (c) studies on the Mollusca fauna in the Hellenic seas [18, 21, 103]. Information from specific web sites was also taken into account (31 March 2017). More specifically, for the species nomenclature update, besides the Marine Biodiversity and Ecosystem Functioning EU Network of Excellence (MarBEF) [58] and the World Register of Marine Species (WoRMS) [104] the Taxonomic on-line Database on European Marine Mollusca (CLEMAM) [34] was used. In addition, the Ellenic Network on Aquatic Invasive Species (ELNAIS) [61] and the Marine Mediterranean Invasive Alien Species database (MAMIAS) [105] were used for the alien species status in the Hellenic and Mediterranean Seas. Records were compared with the checklist of Koukouras [106]. For the molluscan life habits, the Todd databases [107] were taken into account. The specimens are deposited in the premises of the Alexander Technological Educational Institute of Thessaloniki and those of Dr. T. Manousis, C. Kontadakis, G. Polyzoulis and G. Mbazios. Scientists are welcome to have access to the biological material at will and by appointment.
